# Temporary dietary fiber depletion prompts rapid and lasting gut microbiota restructuring in mice

**DOI:** 10.1128/spectrum.01517-24

**Published:** 2025-02-05

**Authors:** Colombe Rous, Julie Cadiou, Hiba Yazbek, Elena Monzel, Mahesh S. Desai, Joel Doré, Maarten van de Guchte, Stanislas Mondot

**Affiliations:** 1 University Paris-Saclay, INRAE, AgroParisTech, Micalis Institute, Jouy-en-Josas, France; 2 Department of Infection and Immunity, Luxembourg Institute of Health, Esch-sur-Alzette, Luxembourg; 3 Faculty of Science, Technology and Medicine, University of Luxembourg, Esch-sur-Alzette, Luxembourg; 4 University Paris-Saclay, INRAE, Metagenopolis, Jouy-en-Josas, France; China Agricultural University, Beijing, China

**Keywords:** dietary fibers, gut microbiota, longitudinal, alternative stable states, mucus, *Muribaculaceae*

## Abstract

Long-term alterations of the gut microbiota and host symbiosis after a dietary perturbation remain insufficiently understood and characterized. In this study, we investigate the impact of temporary dietary fiber depletion in mice that received a diet with reduced fiber content (RFD) for 3 weeks followed by a return to a standard chow diet for 6 weeks, compared to mice that only received a chow diet. Fiber deprivation was accompanied by a reduction of microbiota diversity and an increase in mucolytic and sulfate-reducing bacteria. The activities of enzymes targeting glycans from the host mucus were increased accordingly, while those targeting plant fibers were decreased. On the host side, we report transiently higher quantities of host DNA in feces during the RFD suggesting an impaired gut barrier function. Six weeks after the return to the chow diet, lasting changes in microbiota composition were observed, as exemplified by the replacement of durably depleted amplicon sequence variants close to *Duncaniella dubosii* by other members of the *Muribaculaceae* family. The observation of two distinct gut microbial communities in mice under identical environmental and alimentary conditions at the end of the experiment suggests the existence of alternative stable microbiota states.

**IMPORTANCE:**

In this article, the authors explore the impact of a diet with reduced fiber content on the gut microbiota-host symbiosis in a mouse model. More importantly, they examine the resilience of the intestinal symbiosis after the return to a standard (chow) diet. Some of the measured parameters (intestinal barrier impairment and bacterial glycan-degrading enzymatic activities) returned to control values. However, this was not the case for bacterial richness—the number of different bacteria observed—which remained durably reduced. Among related bacteria, some groups receded and remained undetected until 6 weeks after the return to the chow diet while others saw their abundance increase in replacement. The authors find that a temporary fiber deprivation lasting as little as 3 weeks can cause a transition to an alternative stable microbiota state, i.e., a lasting change in intestinal microbiota composition.

## INTRODUCTION

Dietary fibers have been neglected in Westernized diets even though they have crucial health benefits for humans. Most of these benefits are mediated by the gut microbiota, as these complex polysaccharides cannot be digested or absorbed in the human small intestine. Dietary fibers reach the colon where they are metabolized by gut bacteria expressing a broad array of carbohydrate-active enzymes (1). As many microorganisms in our gut directly or indirectly rely on these dietary fibers for growth (2), the modulation of their availability through diet has a large impact on the composition and activity of the gut microbiota (3). In turn, these changes affect host-microbe interactions and impact their symbiotic relationship.

The microorganisms and the host are in homeostasis, and the gut microbiota is relatively stable over time (4). Recent studies of the human gut microbiota have identified enterotypes (5), discrete ecological configurations of the microbiota (6) that may represent alternative stable states of the human gut microbiota (different states that can exist under identical external conditions) (7) or condition-dependent states. Enterotypes have been associated with long-term dietary habits, while short-term diet changes did not cause a lasting switch in enterotype affiliation (8). Alternative stable states have been documented in pediatric ulcerative colitis patients and linked with their response to anti-inflammatory treatments (9). Characteristic for such alternative states is that the host-microbes equilibrium can be pushed toward a different state, if conditions change beyond the limits of the system’s resilience, and tends to stay in that new state when conditions come back to normal (10). As the first formal demonstration of alternative states in the intestinal ecosystem under controlled conditions, chemically induced colitis in the context of a diet with reduced fiber content (RFD) was proven to induce a switch to an alternative stable state of the host-microbiota ecosystem in rats (11).

**Fig 1:**
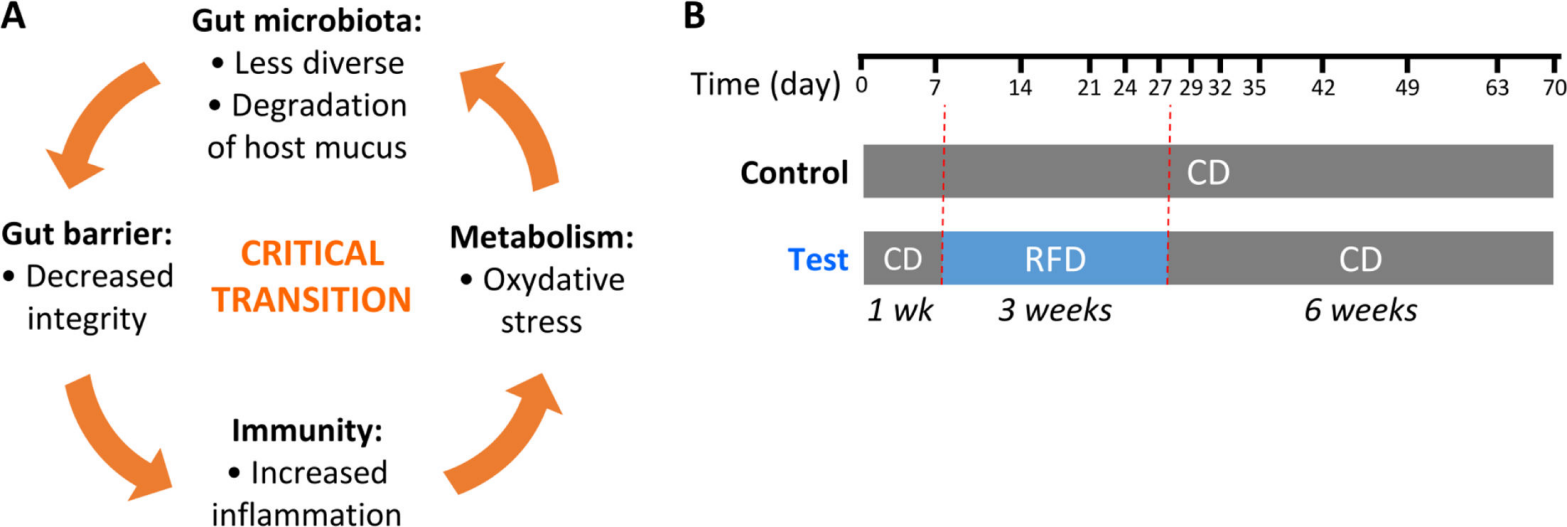
Study hypothesis and design. (**A**) Model of critical transition of the host-microbe system in the context of a reduced-fiber diet. Mutually reinforcing bacteria-host interactions may lead to a state transition of the intestinal ecosystem (see main text for explanation). (**B**) Design of the mouse experiment. The experiment was performed with two groups of five individually housed male mice, 6 weeks old at the beginning of the experiment. The colored bars indicate the different diets (CD: chow diet and RFD: reduced-fiber diet). Time points for feces collection are shown on the timeline.

We hypothesized that the RFD itself could induce a vicious cycle of negative microbiota-host interactions and lead to a state transition of the intestinal ecosystem (Fig. 1A). Gut microbiota associated with fiber-free diets are less diverse (12) and functionally more oriented toward the degradation of host mucins than dietary fibers (13–17). This leads to an erosion of the mucus layer coating the intestinal epithelium, thus threatening the integrity of the intestinal barrier (13, 14, 18). An increased permeability could lead to an over-activation of the host immune system by the gut microbiome and trigger inflammation. The neutrophil-derived inflammation marker lipocalin-2 is notably increased in mice under a fiber-free diet (13, 14). Inflammation triggers oxidative stress (19), which favors deleterious aero-tolerant bacteria that are mostly pro-inflammatory and could sustain if not aggravate inflammation (20). This vicious cycle of alterations could drive a critical transition toward an alternative state in the host-microbe relationship, from which resilience would not be certain.

The resilience of the gut symbiosis following a dietary challenge may be explored insufficiently, using diets that are both low in fiber and high in fat (21, 22) or focusing only on the microbial side of the symbiotic relationship (12). Here, we study the potential resilience of the system after a temporary dietary fiber depletion and evaluate the consequences on both gut microbial composition and host physiology. We observe lasting changes in microbiota composition indicative of a transition to an alternative stable state.

## MATERIALS AND METHODS

### Mice

Six-week-old C57BL/6NRj male mice were purchased from Janvier labs (Le Genest-Saint-Isle, France), hosted at INRAE, Jouy-en-Josas (IERP Unit), and maintained in individual cages under specific pathogen-free conditions with controlled temperature (21°C) and 12 h light/dark cycle. After a week of adaptation to the chow diet (γ-irradiated 25 kGy, Ssniff S8189, Soest, Germany; Table S1), mice were randomly divided in two groups. The first group (« Control »; 5 mice) continued on *ad libitum* chow diet for 9 weeks. The second group (« Test »; 5 mice) was given *ad libitum* RFD (γ-irradiated 25 kGy, Ssniff E15720, Soest, Germany; Table S1) for 3 weeks and chow diet for the following 6 weeks. Litter was changed once per week for all mice, except during the first week following a diet change. Fecal pellets were collected once a week or every day during the week before the first diet switch and during the 3 weeks following the second diet switch and stored at −80°C until use. After euthanasia, colon and liver samples were collected. All animals were included in the analyses.

### Fecal DNA extraction

Fecal DNA extractions were performed with the Nucleospin DNA Stool kit (#740472, Macherey-Nagel, Düren, Germany), following the manufacturer’s recommendation with the following minor modifications. One stool pellet (around 40 mg) was suspended in 850 µL ST1 buffer in an MN Bead Tube Type A and incubated under agitation (600 rpm) at 70°C for 5 minutes. Samples were homogenized using a FastPrep-24 5G (MP Biomedicals, Illkirch-Graffenstaden, France) at 6.5 m/s for 2 minutes. The homogenized samples were incubated a second time at 70°C for 5 minutes under agitation (600 rpm). The fourth wash with ST5 was omitted. DNA was eluted with 50 µL SE buffer. DNA was quantified with a Nanodrop ND-1000 (Thermofisher, Waltham, USA) and kept at −20°C until use.

### Fecal bacterial load estimation

Bacterial load in stool was estimated by real-time qPCR targeting the 16S rRNA coding gene. Fecal DNA was diluted 10,000 times in DNAse-free water, and each reaction was performed in duplicate in a 10 µL volume with 2.5 µL diluted DNA and 7.5 µL reaction mix composed of 1× TaqMan Universal PCR Master Mix (#4304437, Applied Biosystems, Foster City, USA), 0.2 µM forward primer 5′-CGGTGAATACGTTCCCGG-3′, 0.2 µM reverse primer 5′-TACGGCTACCTTGTTACGACTT-3′, and 0.25 µM probe 5′-CTTGTACACACCGCCCGTC-3′. The cycling conditions were the following: 2 minutes at 50°C, 10 minutes at 95°C, and 40 cycles of 15 seconds at 95°C followed by 60 seconds at 60°C. The calibration curve was built as previously described (23), with a pure culture of *Escherichia coli str. K-12 substr. DH10B* (which contains seven copies of the 16S rRNA coding gene) (24).

### 16S rRNA gene sequencing and analysis

The V3–V4 region of the 16S rRNA gene was amplified by PCR in fecal DNA with primers 341F 5′-TACGGRAGGCAGCAG-3′ and 785R 5′-ATCTTACCAGGGTATCTAATCCT-3′ (25), and amplicons were sequenced on a MiSeq sequencer in paired-end mode (2 × 300 bp; Illumina, Munich, Germany) at Eurofins (Nantes, France; samples from time points 7, 21, 27, 35, 42, 49, 63, and 70) or the Integrated Microbiome Resource (Halifax, Canada; samples from time points 14, 24, 29, and 32; see Table S2 for exceptions). Five samples were sequenced at both facilities and compared to check for reproducibility (Fig. S1). Raw sequencing reads were processed with the QIIME2 pipeline (26) (v2021.11.0). Primers were removed, and sequences shorter than 200 bp were eliminated using cutadapt (27) (v2021.11.0). Sequences were truncated to 270 bp for forward reads and 200 bp for reverse reads due to a decrease in quality (median quality score for remaining bases >20 for random sampling of 10,000 sequences), and then denoised, dereplicated, and chimera-filtered to obtain a feature table with amplicon sequence variants (ASVs) using dada2 (28) (v2021.11.0) with default parameters. ASV sequences were annotated using the Naive Bayes feature classifier plugin from QIIME2 (29) with a minimum confidence threshold of 0.7. The classifier was trained on the SILVA reference database (138.1, SSU NR 99) (30) trimmed for the V3-V4 region of the 16S rRNA gene. Additional sequence alignments were performed with BLAST (31) (megablast on 16S rRNA database) to identify species with >99% identity in Fig. 6. Microbial composition analysis was carried out using the ASV count table. The initial distribution of sequencing depth per sample was as follows: mean 23 k reads, maximum 49 k reads, and minimum 5 k reads. ASV counts were therefore adjusted for sample sequencing depth by dividing ASV counts by the total sample read count and multiplying with 5,000. The resulting ASV table was filtered to remove ASVs with less than 10 counts or with a sample prevalence lower than 5. Alpha (Richness, Shannon, and Pielou) and beta (Bray-Curtis dissimilarity, Jaccard index, and Unifrac distance) diversities were analyzed using the vegan (v2.6–4) (32) and GUniFrac (v1.8) (33) packages in R (v4.3.2). Differences between groups were assessed using Fisher-Pitman permutation tests for the α-diversity. Bray Curtis dissimilarity among samples was visualized using either principal coordinate analysis (PCoA, in the case of a limited number of samples) or nonmetric multidimensional scaling (NMDS). The tree used in GUniFrac was constructed with qiime2 (26). ASV sequences were aligned with MAFFT (34) (default parameters). Phylogeny was constructed with FastTree 2 (35) before being mid-point rooted. Analysis of variance using distance matrices was performed with ANOSIM from the vegan package. ALDEx2 (v1.34.0) (36) was used to identify differentially abundant ASV between groups at each time point (after filtering for rare or low-abundance taxa as described just above, no sequencing depth adjustment). Briefly, 200 Monte Carlo instances were sampled from a Dirichlet distribution to determine the probability distribution of the observed data. Afterward, a centered log-ratio transformation (subtraction of the mean from each individual log-transformed value) was applied to the data. Expected *P*-values of Wilcoxon tests were used to compare groups.

### Fecal enzymatic activities determined by p-nitrophenyl glycoside-based enzyme assay

The activities of glycan-degrading enzymes were determined in solubilized fecal proteins as previously described (37) for two enzymes targeting linkages in dietary plant fiber glycans (α-galactosidase and β-glucosidase) and three enzymes targeting host mucus glycans (β-*N*-acetyl-glucosaminidase, α-fucosidase, and sulfatase). Briefly, fecal proteins were extracted by mixing one fecal pellet with 500 µL lysis buffer (50 mM Tris-HCl, 100 mM KCl, 10 mM MgCl_2_, 0.12‰ Triton X-100, DNAse I, lysozyme, and EDTA-free protease inhibitor, and pH 7.25 in ddH_2_O), vortexing and performing pulsed sonication on ice. The samples were then centrifuged, and the supernatant was collected to determine protein concentration with Pierce 660 nm Protein Assay Reagent (#22660, Thermo Scientific, Waltham, USA). A standard curve was created with 4-nitrophenol (#6524.1 Carl Roth, Karlsruhe, Germany). The assay was performed as follows: solubilized fecal proteins were mixed with detection buffer (50 mM Tris-HCl, 100 mM KCl, 10 mM MgCl_2_, and pH 7.25 in ddH_2_O) to equalize the amounts of proteins among samples. 4-Nitrophenyl-coupled substrates were then added to reach the following final concentrations: 10 mM for potassium 4-nitrophenyl sulfate (#N3877, Sigma-Aldrich, Missouri, USA), 4-nitrophenyl *N*-acetyl-β-D-glucosaminide (#N9376 Sigma-Aldrich), and 4-nitrophenyl α-L-fucopyranoside (#N3628, Sigma-Aldrich), and 20 mM for 4-nitrophenyl α-D-galactopyranoside (#N0877, Sigma-Aldrich) and 4-nitrophenyl β-D-glucopyranoside (#N7006, Sigma-Aldrich), which were previously determined as being optimal concentrations (37). The absorption at 405 nm was then measured every 2 minutes at 37°C for 2 hours (or 4 hours for the sulfatase assay). The curve of the detected absorption over time was computed for each sample, and the linear phase with the highest absorption increase per time unit was chosen to perform a linear regression and calculate the slope (absorbance over time) using R (v4.3.1). The enzymatic activity was calculated using the standard curve (absorbance over 4-nitrophenol concentration) to obtain the number of cleaved molecules per unit of time (substrate turnover velocity, unit kat). Finally, this value was normalized on the amount of fecal protein in the sample (unit kat/mg).

### Host DNA quantification in feces

Murine DNA in fecal samples was quantified by real-time qPCR targeting long interspersed nuclear element 1 repeats (38). Fecal DNA was diluted 10,000 times in DNAse-free water, and each reaction was performed in duplicate in a 10 µL volume with 2.5 µL diluted DNA and 7.5 µL reaction mix composed of PowerUp SYBR Master Mix 1× (#A25742, Applied Biosystems, Foster City, USA), 0.2 µM forward primer 5′-AGGCAACGCTGGAGATAGAA-3′, and 0.2 µM reverse primer 5′-ATGCTCGCATCTATGGTTCC-3′. The cycling conditions were the following: 2 minutes at 50°C, 2 minutes at 95°C, and 40 cycles of 15 seconds at 95°C followed by 60 seconds at 60°C. Melting curve: 15 seconds at 95°C, 60 seconds at 60°C, and 15 seconds at 95°C. The temperature ramp increment was 1.6°C/second, except for the melting curve dissociation step for which it was 0.1°C/second. A calibration curve was built with DNA extracted from mouse tissue (DNA extraction protocol according to Yu, Z. & Morrison, M [39]), omitting the purification steps (i.e., digestion with RNase, proteinase K, and use of QIAamp columns), and quantified with a Nanodrop ND-1000 instrument (Thermofisher, Waltham, USA).

### Host gene expression analysis by RT-qPCR

Colon and liver RNA were extracted with TRIzol (#15596026, Invitrogen, Carlsbad, California, USA; safety hazard: toxic, suspected of causing genetic defects) as described previously (40). Reverse transcription was performed according to the manufacturer’s guidelines (High-Capacity cDNA Reverse Transcription Kit, #4368814, Applied Biosystems, Foster City, California, USA), with a starting quantity of RNA of 1 µg. After a 10-fold dilution in DNAse-free water, cDNA was used for qPCR. Each reaction was performed in duplicate, and duplicates whose Ct’s SD was higher than 0.5 were omitted. Reactions were carried out in 10 µL volume with 2.5 µL diluted cDNA and 7.5 µL reaction mix. The reaction mix was composed of PowerUp SYBR Master Mix 1× (#A25742, Applied Biosystems, Foster City, USA), 0.2 µM forward primer, and 0.2 µM reverse primer. The cycling conditions were the following: 2 minutes at 50°C, 2 minutes at 95°C, and 40 cycles of 15 seconds at 95°C followed by 60 seconds at 60°C. Melting curve: 15 seconds at 95°C, 60 seconds at 60°C, and 15 seconds at 95°C. The temperature ramp increment was 1.6°C/second, except for the melting curve dissociation step for which it was 0.1°C/second. Primer couples are described in Table 1 (5′–3′).

**TABLE 1:** RT-qPCR primers used for gene expression analysis

Gene	Gene (full name)	Forward	Reverse	Reference
*Tnfa*	Tumor necrosis factor	CTGTAGCCCACGTCGTAGC	TTGAGATCCATGCCGTTG	Véronique Douard, personal communication
*Muc2*	Mucin 2	CAACAAGCTTCACCACAATCTC	CAGACCAAAAGCAGCAAGGTA	
*ZO1*	Tight junction protein 1	GATCATTCCACGCAGTCTCC	GGCCCCAGGTTTAGACATTC	
*Cldn1*	Claudin 1	CGACTCCTTGCTGAATCTGA	GCCAATGGTGGACACAAAG	
*Cldn2*	Claudin 2	GTAGCCGGAGTCATCCTTTG	GGCCTGGTAGCCATCATAGT	
*Ocln*	Occludin	GCGATCATACCCAGAGTCTTTC	TGCCTGAAGTCATCCACACT	
*Il1b*	Interleukin one beta	AGTTGACGGACCCCAAAAG	AGCTGGATGCTCTCATCAGG	
*Casp1*	Caspase 1	AGATGGCACATTTCCAGGAC	GATCCTCCAGCAGCAACTTC	(41)
*Cat*	Catalase	AGCGACCAGATGAAGCAGTG	TCCGCTCTCTGTCAAAGTGTG	Primer bank ID 6753272a1 (42)
*Lcn2*	Lipocalin 2	AAGGCAGCTTTACGATGTACAGC	CTTGCACATTGTAGCTGTGTACC	(43)
*MCP1*	C-C motif chemokine ligand 2	CTCACCTGCTGCTACTCATTCACT	TTCCTTATTGGGGTCAGCAC	(44)
*Nlrp3*	NLR family, pyrin domain containing 3	ATTACCCGCCCGAGAAAGG	TCGCAGCAAAGATCCACACAG	(45)

### Colon histo-pathological score

Blinded assessment of histo-pathological criteria on colon Swiss rolls (stained with HES after fixation and paraffin embedding) was performed by a pathologist at Excilone (Elancourt, France). The following criteria were graded: mucosal edema, mucosal hyperplasia, atrophia, abscess, squamous metaplasia, polypes, goblet cell hyperplasia, lymphocytes in lymphatic vessel, mucosal, and submucosal mononuclear, and neutrophil cell infiltrates. The severity for each criterion was graded from 0 to 5: 0 meaning absent, 1 minimal/rare, 2 mild/slight, 3 moderate, 4 marked, and 5 severe. The grade (relative to size) and number of GALT loci (gut-associated lymphoid tissue) were also measured.

### Statistical analysis

Statistical significance was assessed by Fisher-Pitman permutation tests on measured values in all instances, except when mentioned otherwise. Effect sizes were estimated with Cohen’s *d*. These analyses were performed using R (v4.3.2), using the coin (v1.4–3) and effect size (v0.8.6) packages, respectively. Correlation analyses were conducted using Pearson’s tests.

## RESULTS

### The reduced-fiber diet reshaped the gut microbiota

In order to investigate the long-term impact of a reduced-fiber diet, we performed an experiment with two groups of five individually housed male mice (Fig. 1B). The test group received a diet with RFD for 3 weeks, followed by a standard chow diet for 6 weeks. The control group received the chow diet for 9 weeks. The RFD contained 9% (wt/wt) dietary fibers as compared to 20% for the chow diet (see Table S1 for more details). Fecal microbiota composition was analyzed longitudinally by sequencing the 16S rRNA coding gene.

**Fig 2:**
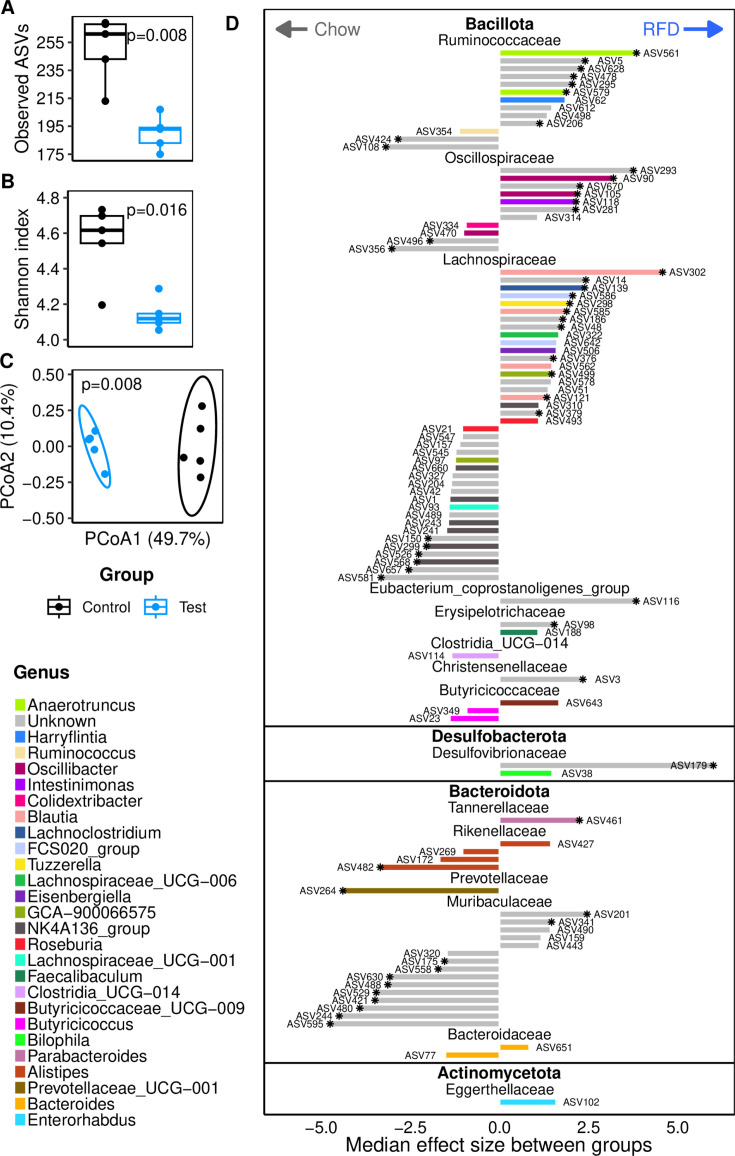
Impact of RFD on gut microbial composition. (**A**) Gut microbiota analysis at the ASV level on day 27. Observed ASVs (richness). (**B**) Shannon index (α diversity). (**C**) PCoA based on Bray-Curtis dissimilarities. Ellipses represent a 95% CI for each group (ANOSIM significance based on a permutation test, 999 permutations). (**D**) Median effect size for relative ASV abundance between groups on day 27. Only including ASVs that were significantly different (Wilcoxon test, no correction, *P* < 0.05; those showing a Wilcoxon *P*-value < 0.1 after Benjamin Hochberg correction are indicated by an asterisk on the corresponding bars). The analysis was conducted using the ALDEx2 (1.20.0) package in R (36). The abundance of these ASVs at day 27 is represented in Fig. S4, and the taxonomic affiliations and statistical data are shown in Table S3. *P*-values from permutation tests.

**Fig 3:**
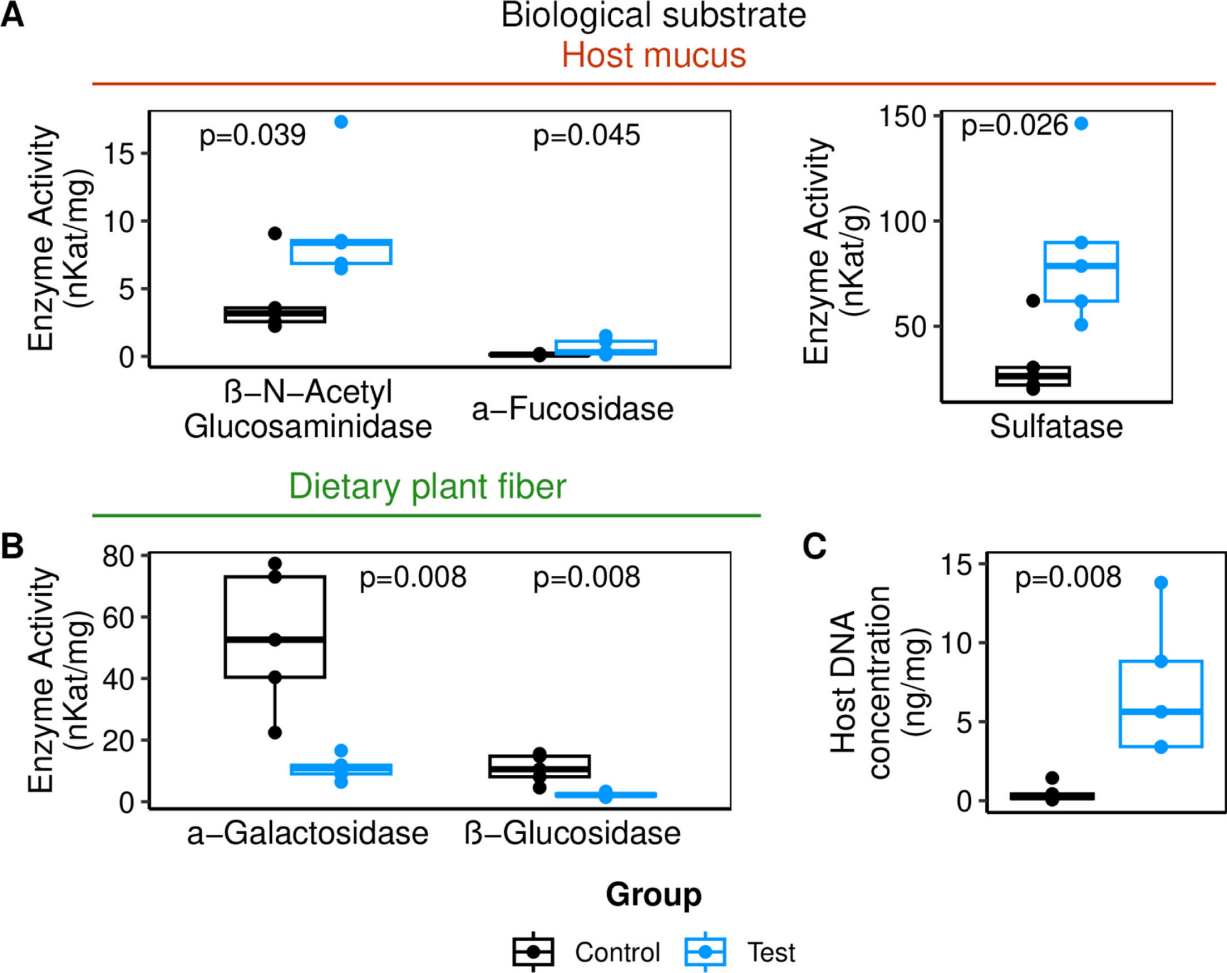
Impact of RFD on gut glycan-degrading capacity and host intestinal barrier. Enzymatic activities in fecal samples at day 25 (2.5 weeks after the beginning of RFD). From a p-nitrophenyl-based assay, expressed as the number of cleaved substrate molecules per second (kat), normalized on the quantity of solubilized fecal protein. For mucus-targeting enzymes (**A**) or plant-fiber-targeting enzymes (**B**). (**C**) Host DNA concentration in feces. *P*-values from permutation tests.

Our first objective was to examine the immediate consequences of a diet with reduced fiber content. During the RFD period, the total quantity of bacteria in feces (as estimated by qPCR on the 16S rRNA gene) was around two times lower in the RFD-fed group than in the Chow-fed group (but only significant at day 21, i.e., 2 weeks into the RFD period, permutation test *P* = 0.024; Fig. S3). On the last day of the RFD (day 27), the richness and the α-diversity of the fecal microbiota were significantly lower in the RFD-fed mice than in the chow-fed mice (Fig. 2A and B). PCoA of microbiota composition showed a clear dissimilarity between the two groups (Fig. 2C). Differential abundance analysis identified 98 ASVs from 15 different bacterial families and 27 genera that were impacted by the RFD when no correction for multiple testing was applied (Wilcoxon test *P* < 0.05), and 20 ASVs from six different families and three known genera (the rest being unknown) after Benjamini-Hochberg correction (Fig. 2D; Fig. S4; Table S3). During the RFD period, the most prominent increase in relative abundance (18-fold) was observed for an ASV from the *Desulfovibrionaceae* family. Otherwise, the majority of the differentially abundant ASVs belonged to the families *Lachnospiraceae* (increased abundance for ASVs in the genera *Blautia* and *Lachnoclostridium*), *Muribaculaceae* (in unknown genera), *Ruminococcaceae* (increased abundance for ASVs in *Anaerotruncus*), and *Oscillospiraceae* (significantly increased abundance for ASVs in *Oscillibacter*). A significant decrease in the abundance of an ASV in the *Prevotellaceae_UCG_001* group was also observed. Given the association of several of the aforementioned genera with a range of glycan degradation activities, we measured bacterial enzymatic activities targeting glycan linkages representative of plant fibers or mucus in fecal samples. The selected plant-fiber-degrading enzymes targeted β-glucosidic linkages (found in plant cell wall) and α-galactosidic linkages (found in oilseed products) associated with a chow diet consumption, while the selected representatives of mucus-degrading enzymes targeted β-*N*-acetylglucosaminide and α-fucosidic linkages, as well as sulfate bonds, commonly found in mucin glycans. The activities of enzymes targeting mucin glycan linkages were increased in RFD-fed mice after 2.5 weeks (Fig. 3A; β-*N*-acetyl-glucosaminidase *P*-value = 0.039 and α-fucosidase *P*-value = 0.045, permutation tests, effect size in Fig. S13). The activity of sulfatase was also significantly increased in the feces of RFD-fed mice (Fig. 3A; *P*-value = 0.026, permutation test). Activities of enzymes targeting dietary fiber polysaccharide linkages were significantly decreased in the feces of mice receiving the reduced-fiber diet (Fig. 3B; *P*-value = 0.008 for both α-galactosidase and β-glucosidase, permutation tests). The two enzymatic activities targeting plant fibers were positively correlated (Pearson’s coefficient 0.99, *P*-value = 1.3 × 10^−16^), as were the activities of enzymes targeting host glycans (β-*N*-acetyl-glucosaminidase – sulfatase: *r* = 0.79, *P* = 0.007; α-fucosidase – sulfatase: *r* = 0.87, *P* = 0.001; β-*N*-acetyl-glucosaminidase – α-fucosidase: *r* = 0.82, *P* = 0.004). Sulfatase activity was inversely correlated with both plant-fiber targeting enzymes (Pearson’s coefficient = −0.66, *P*-value = 0.037). Moreover, activities of enzymes targeting plant fibers were correlated with gut microbiota richness at day 27 (Pearson’s coefficient = 0.84, *P*-value = 0.002 for both α-galactosidase and β-glucosidase).

### Direct impact of the reduced fiber diet on host parameters

No significant differences in weight gain were observed between groups (Fig. S2). Occasional differences were noted in food intake, which indicated a slightly lower intake among the test group during the last week of RFD and 2 weeks following the return to chow (Fig. S2).

Higher amount of murine DNA was detected on day 27 in fecal pellets of RFD-fed mice than in those of chow-fed mice (*P*-value = 0.008, permutation test; Fig. 3C), hinting at an impairment of the gut barrier.

In summary, exposure to a reduced-fiber diet led to a profound change in microbiota composition that favors mucolytic activity and a presumed consequent weakening of the intestinal barrier.

### The reduced-fiber diet had a lasting impact on the gut microbiota

Our second objective was to characterize the resilience, or lack thereof, of the host-gut microbiota symbiosis after the temporary reduced-fiber diet. For that purpose, mice were switched back to a chow diet for 6 weeks at the end of the RFD period, and the microbiota was sampled at different time points to study post-perturbation microbial dynamics (Fig. 1B). The control group was kept on the chow diet during that same period.

**Fig 4:**
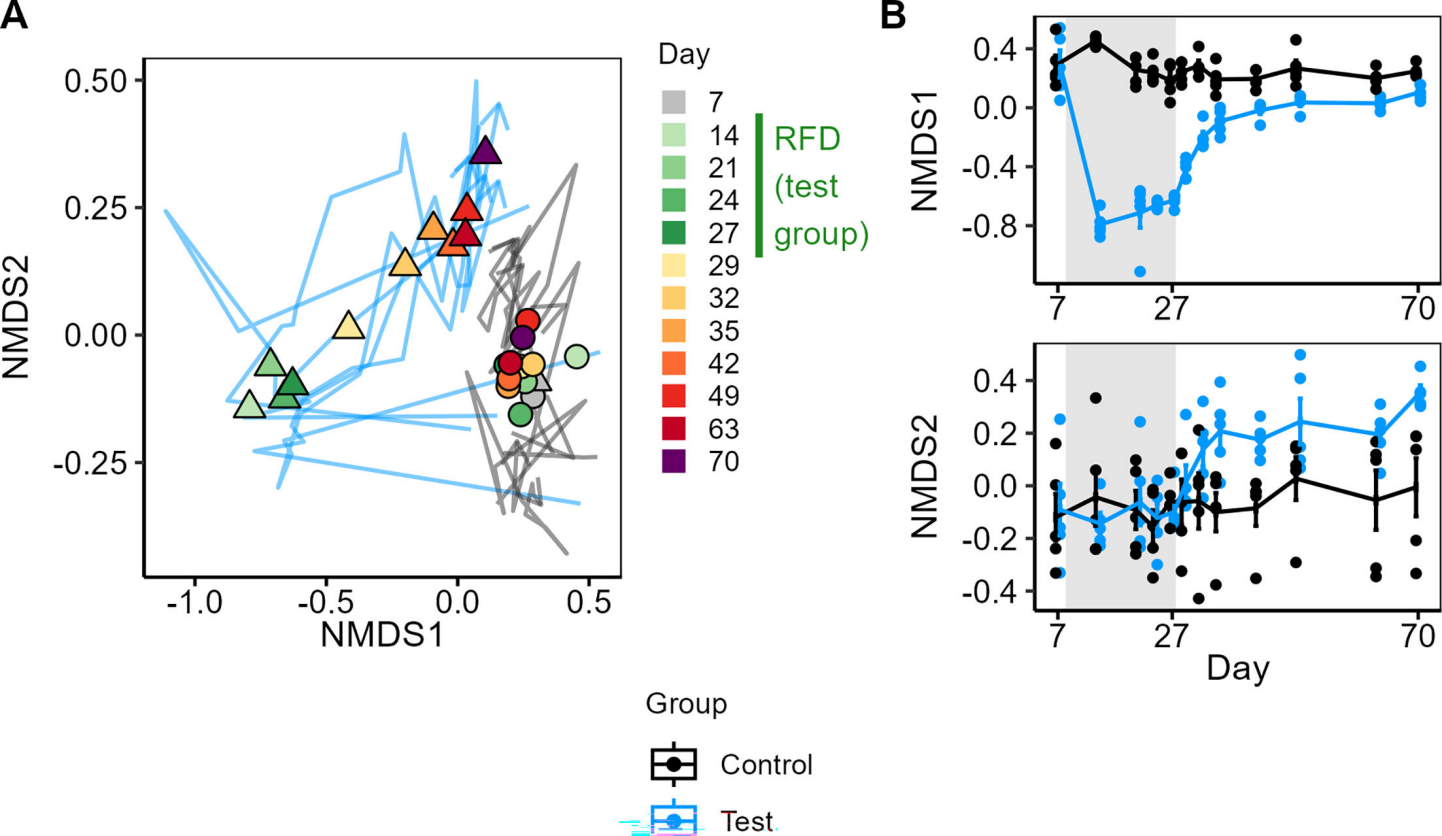
Dynamics of global gut bacterial composition during and after the RFD. (**A**) Non-metric multidimensional scaling (NMDS) of gut microbiota composition over time, based on ASV-level Bray-Curtis dissimilarities. Each point represents the centroid of a group (test ▲ or control ●) at a given day, as indicated by the color scale (gray before stress, green during RFD [for the test group], and orange/red/purple during the recovery period), and each trajectory represents one mouse during the experiment (in blue for the test group and gray for the control group). (**B**) Gut microbiota dynamics on first and second axes of NMDS. The RFD period (test group) is represented by the gray area. Error bars represent SEM, and lines connect group means at each time point.

**Fig 5:**
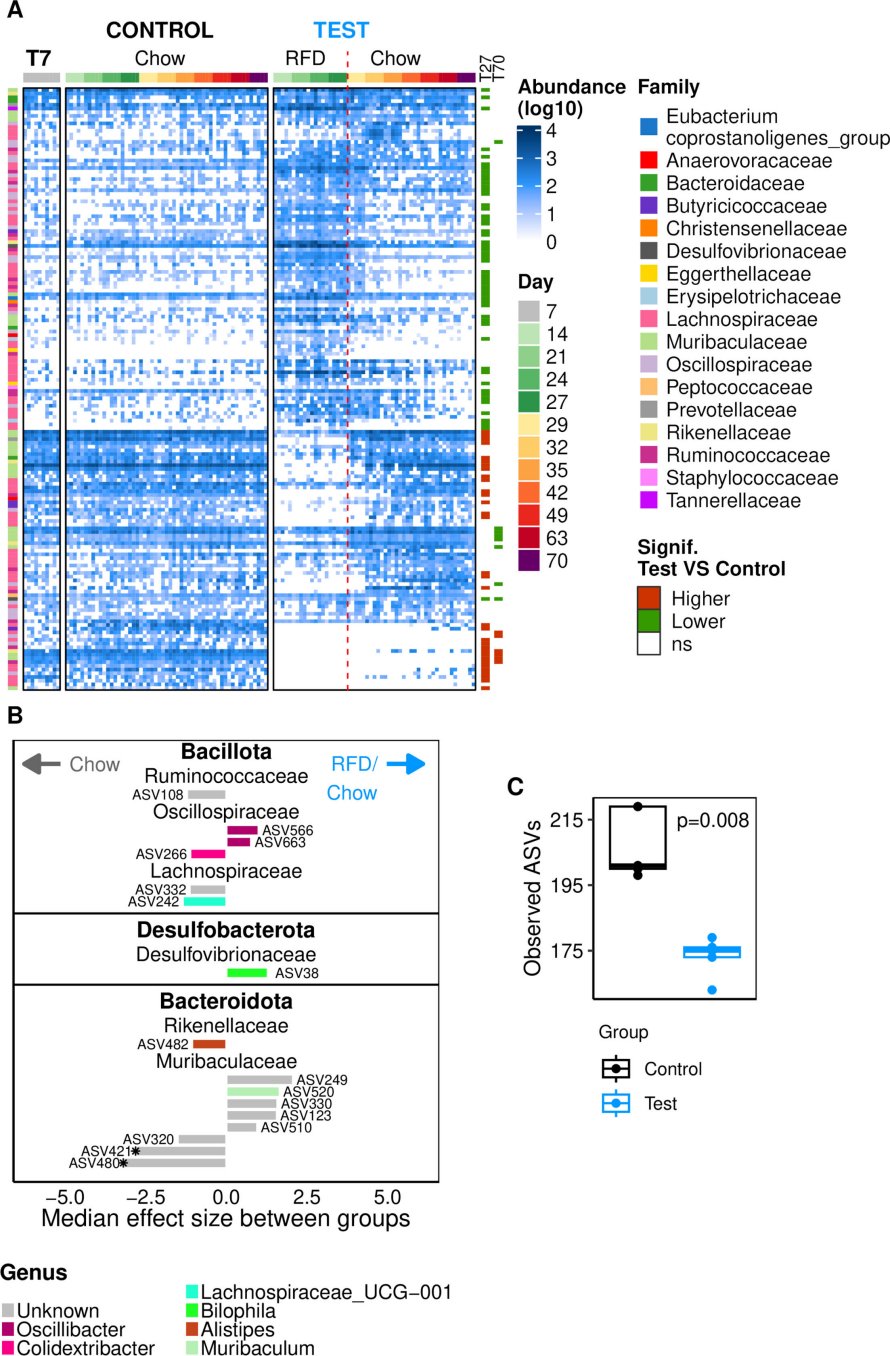
Lasting impact of the RFD on gut bacterial composition at the ASV level. (**A**) Normalized ASV read counts heatmap (log_10_ transformed) of ASVs that were differentially abundant between groups at least at one time point (from ALDEx2 analysis, *P* value from a Wilcoxon test <0.05, no correction). Each row represents one ASV. Each column represents one mouse (in the same order for each time point). The “T7” block includes five mice from the control group followed by five mice from the test group before any treatment (day 7). The dotted red line represents the end of the RFD and the beginning of the chow diet for the test group. Columns T27 and T70 highlight ASVs that were differentially abundant between groups at the end of RFD (day 27) or at the end of the experiment (day 70), respectively. Constructed using the ComplexHeatmap package (v2.18.0) (46). (**B**) Median effect size for ASV abundance between groups on day 70. Only including ASVs that were significantly different (Wilcoxon test, *P* < 0.05, no correction; those showing a Wilcoxon *P*-value < 0.1 after Benjamin Hochberg correction are indicated by an asterisk on the corresponding bars). The analysis was conducted using the ALDEx2 (1.34.0) package in R (36). The abundance of these ASVs at day 70 is represented in Fig. S6, and the taxonomic affiliations and statistical data are shown in Table S4. (**C**) Observed ASVs (richness) on day 70.

NMDS (Fig. 4A) analysis showed that the RFD had induced a large deviation in microbiota composition as soon as 7 days after the start of this diet, which remained stable afterward. After shifting back to the chow diet, the microbiota rapidly evolved again to a new state within a week and remained nearly constant over the next month. This new state did not correspond to the original state before the RFD intervention or the state of the control group at the same time. On NMDS axis 1 (Fig. 4B), while approaching its initial state, the test group remained at a distance from the control group at day 70. On axis 2 in contrast (Fig. 4B), a large deviation from the initial position and that of the control group rapidly built up only after discontinuation of the RFD and stabilized thereafter. The two groups had a significantly different gut microbiota composition at day 70, as established by an analysis of variance using distance matrices (ANOSIM) with Bray-Curtis dissimilarities (*P* = 0.03), Jaccard index (*P* = 0.03), and generalized or unweighted UniFrac (*P* = 0.01 and 0.02, respectively). Group differences were not significant, however, when using weighted UniFrac distances (*P* = 0.1), which could indicate that microbiome deviations are for a large part found in less abundant ASVs. Figure 5A presents a heatmap of all the ASVs that were differentially abundant between groups on at least 1 day of the experiment. The top of the heatmap depicts ASV whose relative abundance was temporarily increased during the RFD period. The bottom shows ASVs that were less abundant during the RFD. Finally, the last rows of the heatmap clearly show ASVs that dropped below detection levels during the RFD and remained undetectable in the majority of the mice following the return to chow. For some of these ASVs, abundance remained below the detection threshold in all five mice at the end of the experiment. Due to the limited population size of five mice per group, statistical testing of differential ASV abundance between groups is very constraining. Therefore, even though 67 ASVs had an average abundance that was either five times higher or five times lower in the test group compared to the control group, only 16 ASVs exhibited significantly different abundance levels (Wilcoxon test, *P* < 0.05) when no correction for multiple testing was applied and none after Benjamini-Hochberg correction (Fig. 5B). Eight of the differentially abundant ASVs (no correction) were more abundant in the test group, while eight were more abundant in the control group (Fig. 5B; Fig. S5; Table S3). Of the eight ASVs with a reduced abundance in the test group on day 70, seven strongly decreased during the RFD period and remained strikingly lower than in the control group in all sampling points during the return to a chow diet (most of them were durably undetected). Five of these appear in Fig. 5A with a significantly lower abundance after RFD on day 27 (ASVs 108, 320, 421, 480, and 482), while the other two also had a lower abundance but not significantly (ASVs 266 and 242). The abundance of the eighth ASV exhibited a fluctuating pattern, declining 2 weeks after the return to chow to reach a lower level than that of the control group. The eight ASVs that were more abundant in the test group belonged to the genera *Oscillibacter* and *Bilophila* or were uncultured *Muribaculaceae*. Both ASVs from *Bilophila* and *Oscillibacter* already exhibited an increased relative abundance after the RFD. On the contrary, the *Muribaculaceae* ASVs with an elevated abundance in the test group following the return to chow displayed a lower abundance during the RFD. These results show that the relative abundance of sixteen ASVs was durably affected by the RFD. Moreover, gut microbiota richness remained significantly lower in the test group than in the control group at the end of the experiment (Fig. 5C; Fig. S6 for longitudinal analysis). This was not the case for α-diversity (Shannon index) or evenness (Pielou index; Fig. S6, effect size in Fig. S13), which returned to control values and subsequently remained relatively stable.

Differences in plant fiber- and mucin-degrading enzymatic activities observed in feces at day 25 (during the third week of RFD) diminished rapidly 3 days after returning to the chow diet (day 30) and were no longer visible after 4 weeks (day 56; Fig. S7). The lasting changes in gut bacterial abundances were thus not associated with the enzymatic activities measured at the end of the experiment.

### The reduced-fiber diet provoked durable changes in the *Muribaculaceae* family

**Fig 6:**
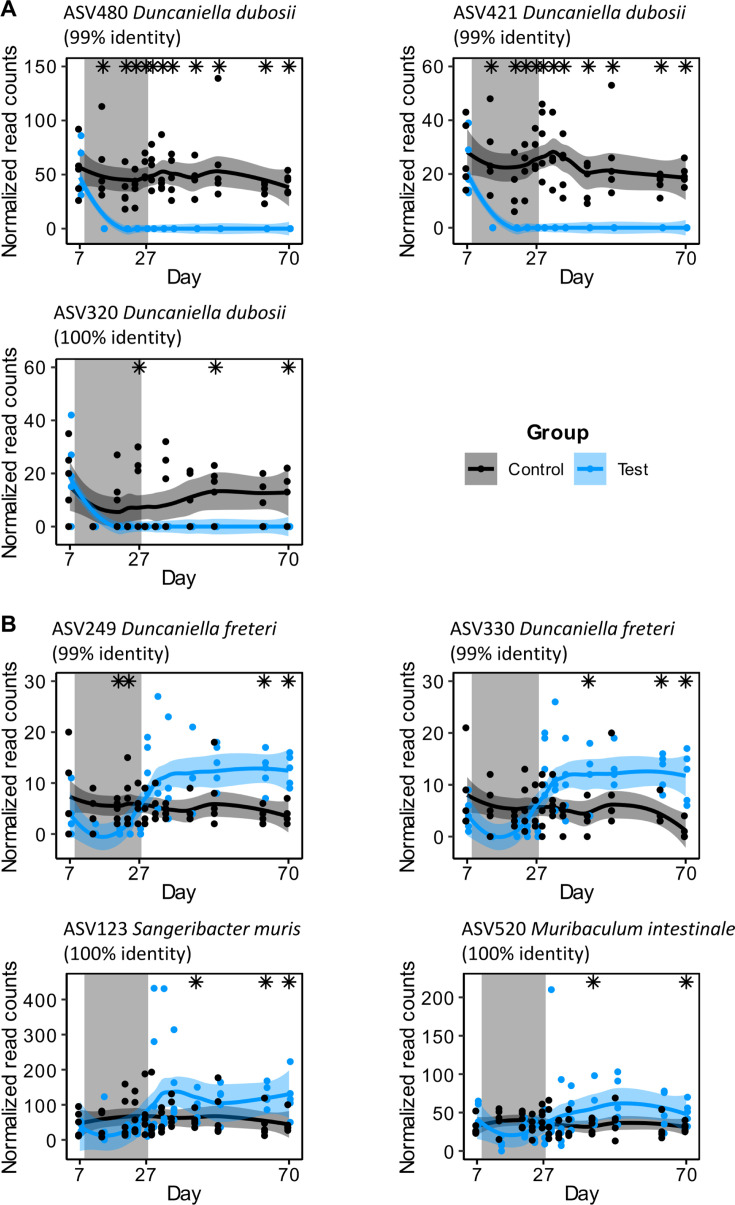
Lasting changes in the *Muribaculaceae* family following RFD. (**A**) Normalized read counts of ASVs belonging to the family *Muribaculaceae* were not detected during the RFD and until the end of the experiment. Stars represent significant differences between groups (Wilcoxon rank test, *P* < 0.05). Normalized read counts refer to the counts after correction for sequencing depth, see Material and Methods for more details. (**B**) Read counts of ASVs identified as *Muribaculaceae* that have an increasing abundance during the post-perturbation period and reach levels that are higher than in the chow group. Significant differences between groups were observed for all ASVs at day 70 (Wilcoxon rank test, *P* < 0.01). Smooth curves were fitted with the LOESS method, the shaded area represents a 95% CI. The gray area on the “day” axis represents the RFD period (test group). Percentage identity from BLAST (31).

The *Muribaculaceae* family accounted for 69 ASVs in our sequencing data set and had a relative abundance of around 30% among the samples in the control group, at all time points. On the last day of the experiment, 8 out of the 16 significantly different ASVs belonged to the *Muribaculaceae* family. This led us to study the dynamics of this family during and following the RFD perturbation. The family as a whole was significantly less abundant during the RFD period (19.0% in all samples from the test group at day 27 vs 34.3% in the control group) but recovered to a mean abundance that was slightly higher than in the control group (30.2% in all test samples at day 70 vs 26.6% in the control group) after return to the chow diet (Fig. S8). Three ASVs from this family, phylogenetically close to the species *Duncaniella dubosii* (phylogenetic tree in Fig. S9) with relative abundances of 0.4%, 0.5%, and 1% in all samples before the RFD period became undetectable during and after the RFD (Fig. 6A). Interestingly, four other ASVs from this family, close to species *Duncaniella freteri* (x2), *Sangeribacter muris*, and *Muribaculum intestinale* (phylogenetic tree in Fig. S9), with relative abundances of 0.12%, 0.13%, 0.83%, and 0.81% before RFD, exhibited a higher abundance than the control group at the end of the experiment (0.26%, 0.24%, 2.55%, and 1.02%, respectively, for the test group, and 0.08%, 0.02%, 0.98%, and 0.64%, respectively, for the control group). As illustrated in Fig. 6B, the relative abundance of these ASVs decreased during RFD, likely due to their sensitivity to the reduced-fiber diet. However, it rapidly increased following the return to chow, reaching levels that exceeded those of the controls. This shows that the RFD had a lasting impact on the *Muribaculaceae* ecology.

### No measurable long-term effect of the RFD on host parameters

As durable changes caused by the RFD were identified in the gut microbiota, we then examined if a lasting impact on the host and the overall equilibrium between the two members of the symbiosis could be observed. We first looked at inflammation markers in organs collected on the last day of the experiment, expecting low-grade inflammation if any. Inflammation-related gene expression in the proximal colon and liver (*Caspase-1*, *Hif1a*, *Cat*, *Lcn2*, *Nlrp3*, *MCP-1*, *Tnfa*, and *Il1b*) studied with RT-qPCR (Fig. S10, effect size in Fig. S13) did not reveal significant differences between groups, except for the expression of lipocalin 2 (*Lcn2*), which was lower in the colon of the test group than in the control group. Histo-pathological scoring of inflammation-related symptoms in the distal colon revealed no significant differences between the two groups (Fig. S11). Regarding intestinal barrier function, gene expression analysis in the proximal colon (*ZO1*, *Ocln*, *Cldn 1*, *Cldn 2*, and *Muc2*) did not show significant differences between groups (Fig. S10). The murine DNA concentration in stool was not significantly higher in the test group than in the control group at the end of the experiment, on day 70 (Fig. S12, effect size in Fig. S13).

## DISCUSSION

While diet is known to greatly affect the microbial communities colonizing the gut (12–15), the lasting effects of temporary diet changes are much less studied. Here, we examined the impact of temporary fiber deprivation in mice, mimicking an exposure to the reduced fiber content of a Western diet, followed by the return to a normal diet (i.e., a chow diet for mice) and the dynamics of microbiota alteration. As a direct effect of fiber deprivation, we observed a rapid change in microbiota composition, with the establishment of a new equilibrium characterized by reduced bacterial load, richness, and diversity within 1 week. At the end of the 3-week fiber deprivation period, a clear disruption of the gut microbiota composition was observed. The abundance of the *Prevotellaceae_UCG-001* genus was decreased after the RFD, coherent with the common association of *Prevotella* with diets rich in fibers (8). Similarly, two ASVs from the genus *Butyricicoccus* were decreased following RFD. These are close to *Butyricicoccus* species that are known to produce butyrate from fiber fermentation (47). Conversely, the RFD-fed group showed an increased abundance of ASVs belonging to the *Lachnoclostridium*, *Anaerotruncus*, and *Eisenbergiella* genera, which have been associated with mucus degradation (48, 49). Several uncultured members of the family *Muribaculaceae* were also enriched in the RFD-fed group, while the abundance of others decreased. This may be explained by the fact that some members of this taxon have been described as mucin monosaccharide foragers (50), while others have been described as plant-fiber-degrading bacteria (51). Similar observations were made for ASVs of the genera *Alistipes* and *Bacteroides*, which demonstrated heterogeneous responses to the RFD. Some representatives of these genera have been shown to grow on mucin (49, 50). In summary, the observed shift in gut microbiota composition pointed to an increased relative abundance of mucolytic bacteria and a decreased abundance of dietary fiber-degrading bacteria, in line with earlier observations (13, 14). This observation was corroborated by the quantification of enzymatic activities in feces, revealing an increase in mucolytic activity and a decrease in fiber-metabolic activity. Other studies have demonstrated that under similar conditions, a metabolic shift in generalist bacteria, which was not studied here, could also contribute to the altered enzymatic activity profiles (13).

In both our and other studies (13, 14), an increase in sulfate-reducing bacteria (SRB) from the *Desulfovibrionaceae* family was observed during fiber depletion. Mucin hydrolysis releases monosaccharides and free sulfate. The latter can be used by SRB as an electron acceptor in anaerobic respiration to form hydrogen sulfide (H_2_S) (52, 53), a potent reducing agent that reduces disulfide bonds that maintain the mucus network integrity. Consequently, the mucus becomes more permeable and accessible for bacteria that can degrade it, creating a vicious cycle of mutually reinforcing activities that promote the erosion of the intestinal barrier (53). We observed an increase in the concentration of host DNA in feces after RFD, indirectly pointing at a degradation or dysfunction of the gut barrier.

After a second diet shift, returning to a chow diet, a new microbiota composition was again reached within 1 week and remained stable for the remaining 5 weeks of the experiment. The new microbiota composition clearly distinguishes itself from the original composition, before fiber deprivation (Fig. 4A). Importantly, it also clearly differs from that of the control group at the same time point, under identical controlled external and dietary conditions, and can thus be considered an alternative microbiota state (11). A detailed analysis revealed noticeable differences between the new and the control microbiota states. First, a replacement of taxa in the *Muribaculaceae* family (Fig. 6) that are most likely involved, directly or indirectly, in fiber degradation. The replacement of *Duncaniella dubosii* members, which remained undetected after the RFD, with other *Muribaculaceae* taxa likely contributed to maintaining fiber-degradation functions (as indicated by the rapid recovery of measured fiber-degrading enzyme levels following the return to chow). However, since the replacement involved different taxa, this might indicate changes in other functions inherent to the amplified taxa, which were not studied here. Second, a lower richness (Fig. 5C) was observed in the new microbiota, a feature which in a more extreme form is generally associated with compromised health (54). Finally, differences in relative abundances of less abundant bacteria (as suggested by the difference between weighted and unweighted UniFrac analysis results), as well as more abundant ones (in the *Muribaculaceae* family) were identified. Among the ASVs that were differentially abundant between groups after the period of return to chow, one was associated with *Bilophila*, a member of the sulfate-reducing *Desulfovibrionaceae* which has been associated with gut barrier impairment (55). Additionally, the relative abundance of ASVs in the *Oscillibacter* genus was increased during RFD and remained higher compared to the control group until the end of the experiment. Although less characterized, this genus has been negatively correlated with transepithelial resistance of the proximal colon (56), thereby favoring permeability. A lasting reduction of microbiota richness after temporary exposition to an RFD has earlier been reported in (humanized) mice (12) and even shown to lead to species extinction over generations. Here, in addition, we show more dynamic detail (Fig. 4A) and taxa replacement. As indicated by the placement of points on both axes of the NMDS analysis during the period of return to chow and the alpha-diversity measurements, the gut microbiota of mice having received the RFD appear to be relatively stable from 1 week after the end of the RFD. However, an extended period of observation would be required to ascertain the long-term stability of this new state. We did not observe any significant lasting effects of the RFD on host parameters, with the exception of a reduction in lipocalin 2 gene expression. In previous studies, the consumption of an RFD was accompanied by an increased intestinal inflammatory tone, a higher susceptibility to pathogen invasion in conventional mice (13, 14), and an increased colitis severity in genetically susceptible mice (57). In our study, end-point measurements were performed 6 weeks after the test group had returned to the chow diet. It is possible that lasting changes in the gut microbiota were not accompanied by durable alterations in the host barrier and inflammatory status. Alternatively, the levels were too low to be detectable, and the identification of lasting scars of the RFD on the intestinal symbiosis requires additional perturbation of the system. Additionally, the small portion of fibers (9%) remaining in the RFD (from corn starch, maltodextrin, and cellulose) may have been used by certain bacteria, thereby facilitating their recovery or the recovery of the whole community, after the return to the chow diet. Finally, a longer period of dietary fiber depletion could have disturbed the symbiosis more profoundly, potentially leading to more damage on the host side of the relationship. Increasing the duration of the RFD period would be justified when considering human populations, which often adopt a fiber-poor diet for extended periods of time, or throughout their entire life.

In conclusion, we provide proof of the principle that temporary fiber deprivation for a period as short as three weeks, and possibly even 1 week (Fig. 4A), can lead to a transition to an alternative stable microbiota state. Alternative state properties complicate the return to the previous state when re-establishing environmental (including dietary) conditions (7). Even if not directly deleterious, this alternative state with a decreased richness may predispose the individual to intestinal barrier degradation and disease development upon a second challenge (for example after infection). If translatable to humans, this knowledge should stimulate preventive action, in the form of a healthy diet, in order to prevent transition to such an alternative state.

## Data Availability

The 16S rRNA sequencing data is available at NCBI BioProject ID PRJNA1077886.

## SUPPLEMENTAL MATERIAL

Supplemental figures spectrum.01517-24-s0001.docx Fig. S1 to S13.

Table S1 spectrum.01517-24-s0002.xlsx Diet descriptions.

Table S2 spectrum.01517-24-s0003.xlsx Sequencing facilities details.

Table S3 spectrum.01517-24-s0004.xlsx List of ASVs with significantly different abundances between groups after the RFD (day 27).

Table S4 spectrum.01517-24-s0005.xlsx List of ASVs with significantly different abundances between groups following the return to chow (day 70).

ASM does not own the copyrights to Supplemental Material that may be linked to, or accessed through, an article. The authors have granted ASM a non-exclusive, world-wide license to publish the Supplemental Material files. Please contact the corresponding author directly for reuse.
