# Supplementary material for: Temporary dietary fiber depletion prompts rapid and lasting gut microbiota restructuring in mice
Source: Microbiol Spectr. 2025 Feb 5;13(3):e01517-24. doi: 10.1128/spectrum.01517-24 (PMC11878010; doi:10.1128/spectrum.01517-24)
Supplement: Supplemental figures — Fig. S1 to S13. [file spectrum.01517-24-s0001.docx]

**Temporary dietary fiber depletion prompts a rapid and lasting gut microbiota restructuration in mice**

Colombe Rous^a^, Julie Cadiou^a^, Hiba Yazbek^a^, Elena Monzel^b,c^, Mahesh S. Desai^b^, Joel Doré^a,d^, Maarten van de Guchte^a^, Stanislas Mondot^a*^

^a^ University Paris-Saclay, INRAE, AgroParisTech, Micalis Institute, Jouy-en-Josas, France

^b^ Department of Infection and Immunity, Luxembourg Institute of Health, Esch-sur-Alzette, Luxembourg

^c^ Faculty of Science, Technology and Medicine, University of Luxembourg, Esch-sur-Alzette, Luxembourg

^d^ University Paris-Saclay, INRAE, Metagenopolis, Jouy-en-Josas, France

* [stanislas.mondot@inrae.fr](mailto:stanislas.mondot@inrae.fr), Domaine de Vilvert, Jouy-en-Josas, France

(a)

(b)
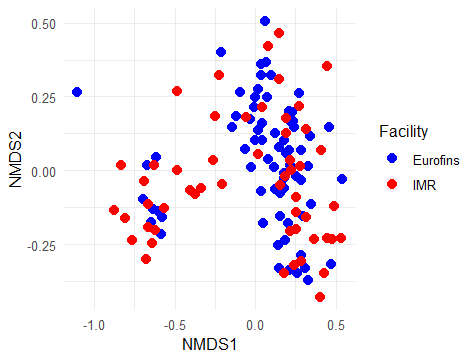


**Supplementary Fig. S1. Sequencing reproducibility check.** (a) Genus counts (log10) for samples that were sequenced at both facilities, all p-values for linear regression were < 2e-16. BC: Bray-Curtis dissimilarities between replicates. (b) Non-Metric Multidimensional Scaling on Bray-Curtis dissimilarities of all sequenced samples, colored by sequencing facility.


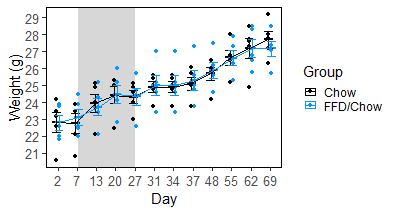

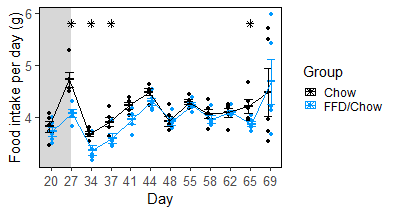


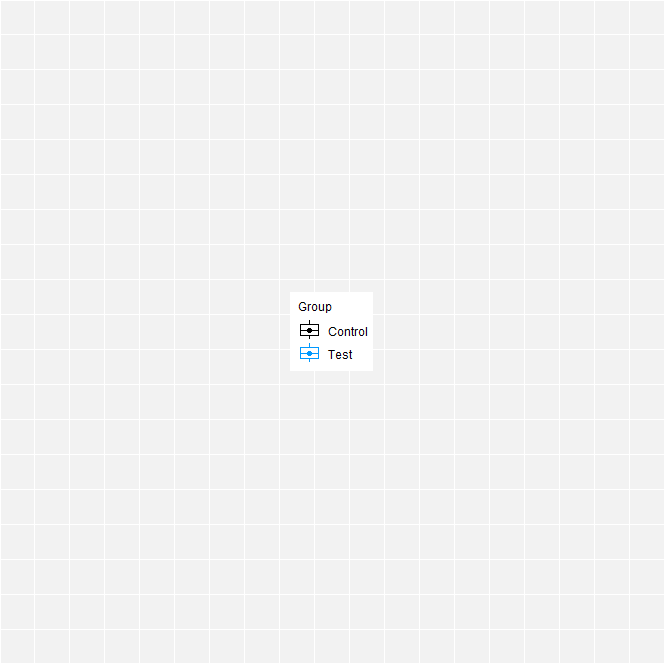


**Supplementary Fig. S2. Weight and food intake across time. ⚹**: p<0.05, permutation test. The grey area represents the RFD (test group).


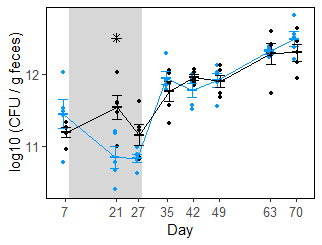

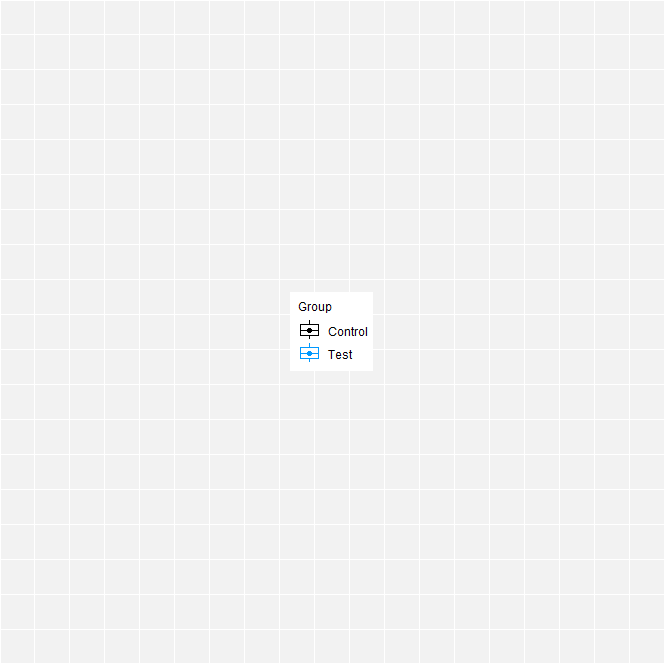


**Supplementary Fig. S3. Fecal bacterial load estimation.** Quantification of the gene coding for 16S rRNA by qPCR in DNA extracted from feces, estimation based on a standard curve constructed with DNA from a pure *E. coli* culture. **⚹**: p<0.05, permutation test. The grey area represents the RFD (test group).


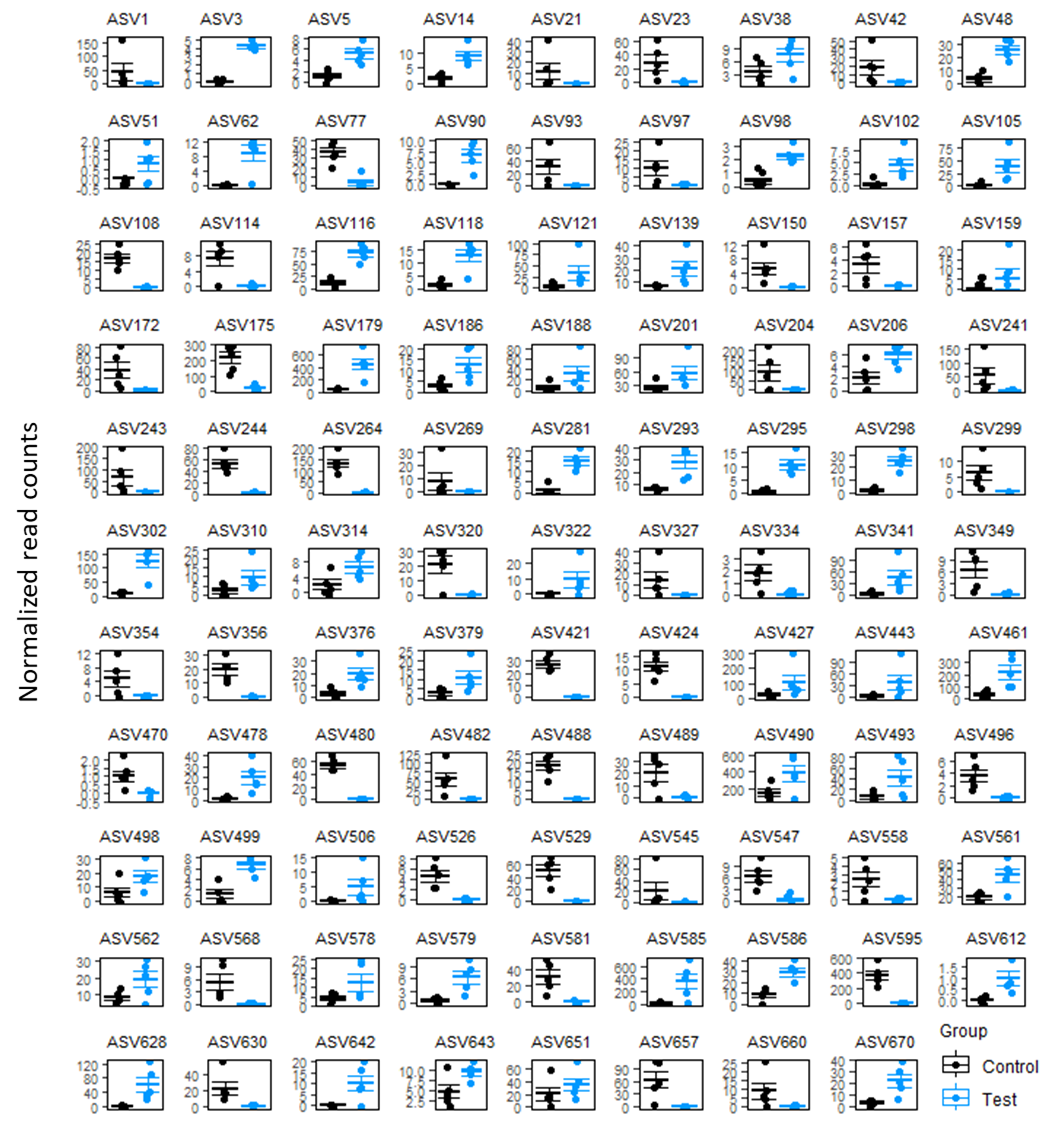


**Supplementary Fig. S4. Normalized read counts for ASVs that are significantly different between groups after RFD (at day 27)**. From ALDEx2 analysis, Wilcoxon p-value < 0.05, no correction. Error bars represent the s.e.m.


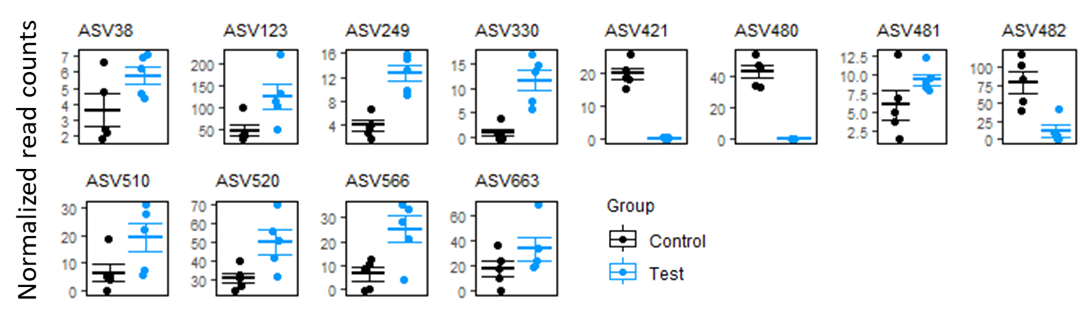


**Supplementary Fig. S5. Normalized read counts for ASVs that are significantly different between groups at the end of the experiment (at day 70)**. From ALDEx2 analysis, Wilcoxon p-value < 0.05, not corrected. Error bars represent the s.e.m.


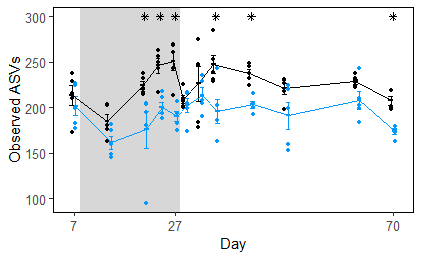

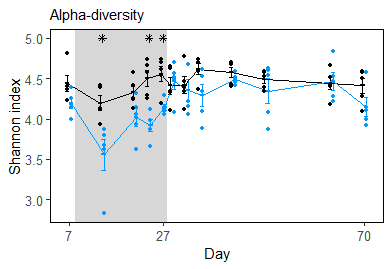


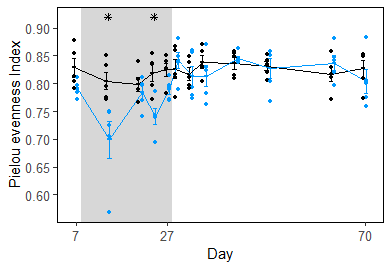

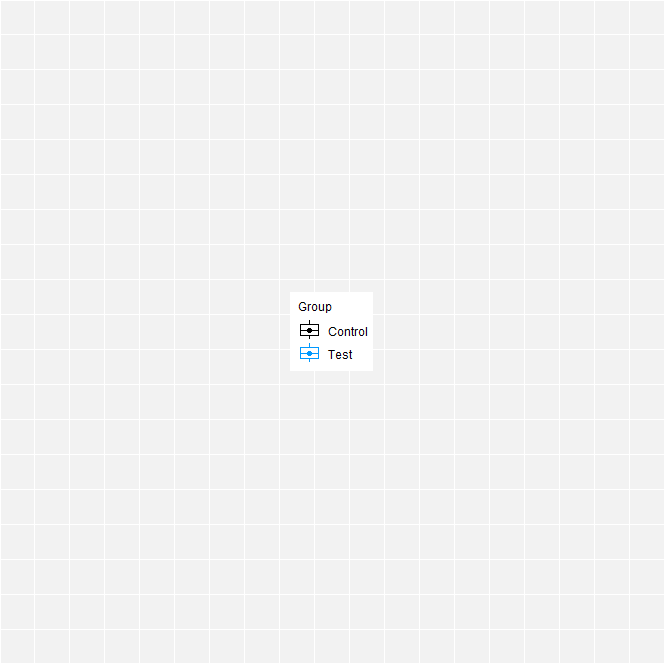


**Supplementary Fig. S6. Longitudinal analysis of gut microbial diversity across time.** Richness (observed ASVs), α-diversity (Shannon index) and evenness (Pielou index). **⚹**: p<0.05, permutation test. The grey area represents the RFD (test group).


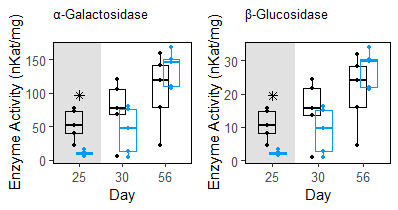

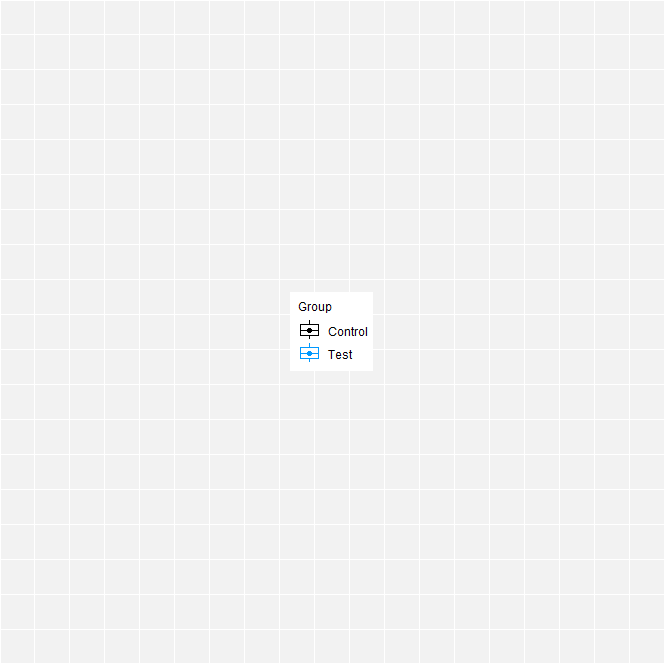


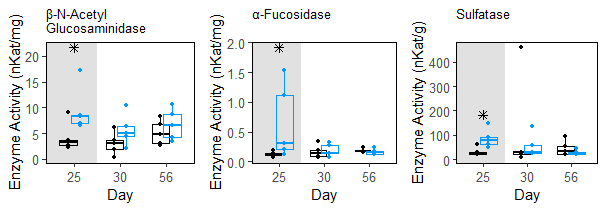


**Supplementary Fig. S7. Bacterial glycan-degrading enzymatic activities in feces across time (during and after RFD).** Measured with a p-nitrophenyl-glycoside based assay. **⚹**: p<0.05, permutation test. The grey area represents the RFD (for test group).


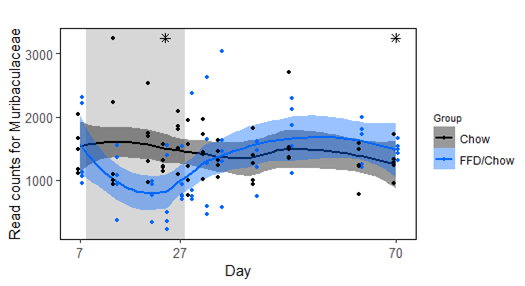

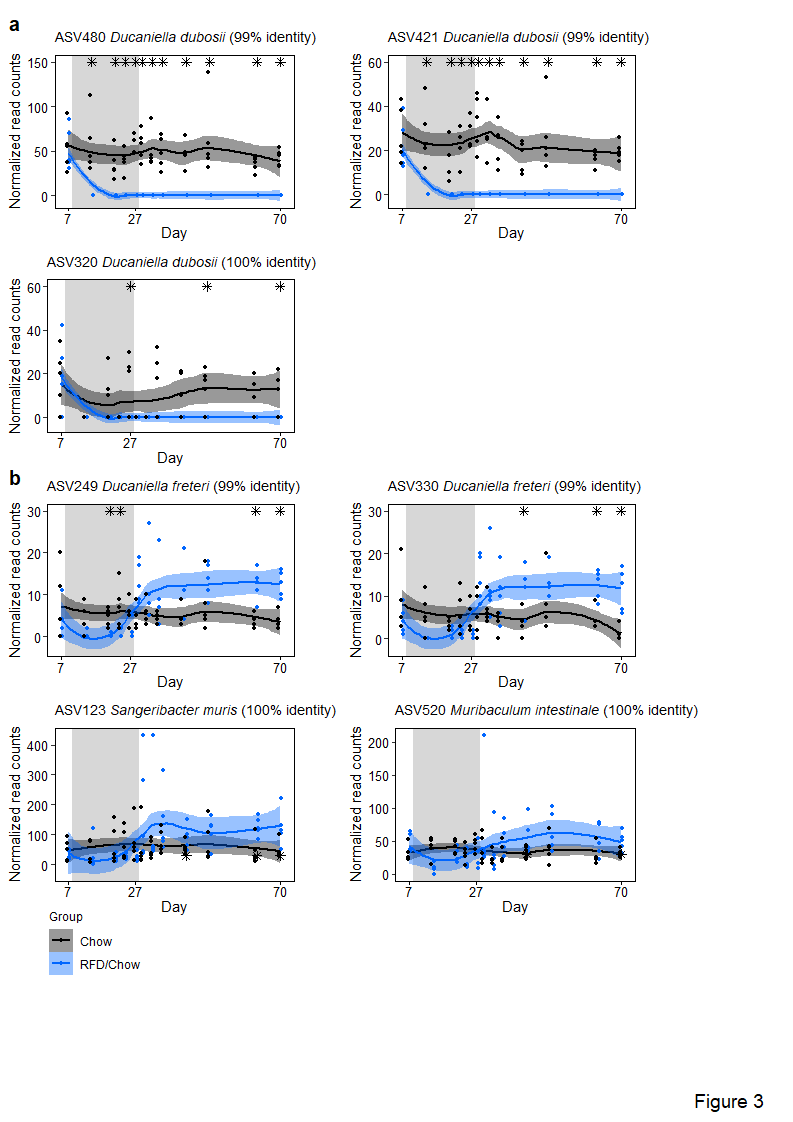


**Supplementary Fig. S8. Total abundance of members from the *Muribaculaceae* family in feces across time. ⚹**: p<0.05, Wilcoxon test with ALDEx2 pipeline ^1^. Smooth curves were fitted with loess method, the shaded areas represent a 95% confidence interval. The grey area on the “Day” axis represents the RFD period (test group).

**
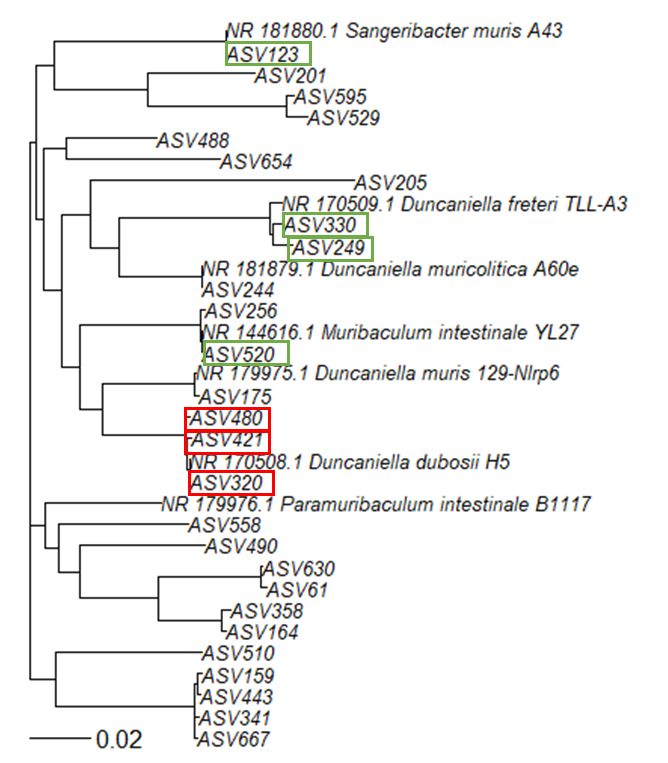
**

**Supplementary Fig. S9. Phylogenetic tree of *Muribaculaceae* ASVs.** Showing those that were significantly different between groups at least at one time point (Wilcoxon test without correction using the ALDEx2 pipeline^1^), as well as reference sequences^2–8^. Sequences were aligned using ClustalOmega. Pairwise distances from DNA sequences were computed using the K80 evolutionary model. The tree was constructed with the neighbor joining method. These steps were performed using the ape package^9^ (v5.8) on R. Red boxes surround ASVs that were less abundant in the test group compared to the control group at the end of the experiment, and green boxes those that were more abundant.

**(a) In colon**

**(b) In liver**


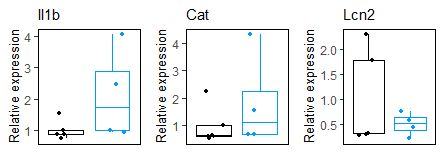

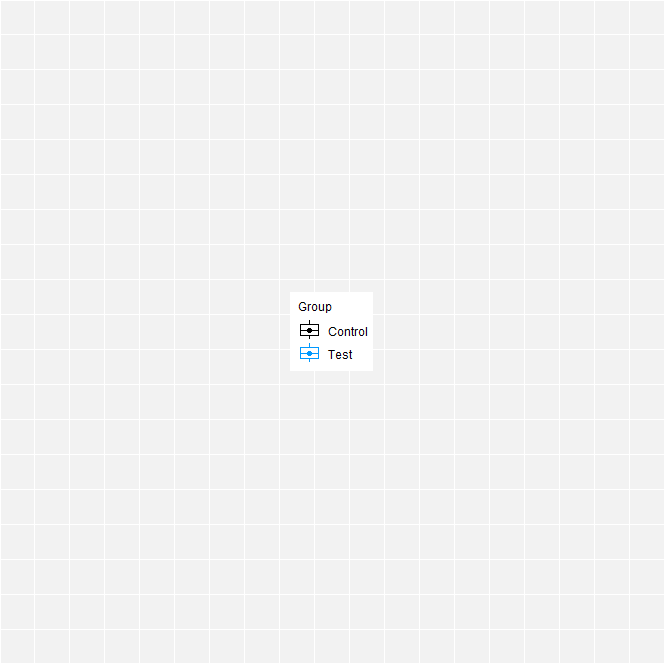


**Supplementary Fig. S10. Assessment of inflammation and intestinal barrier integrity.** Relative expression of marker genes at the end of the experiment, measured by RTqPCR and normalized on the mean expression of the control group. P-value calculated with a Fisher-Pitman permutation test, only displayed if p<0.05.


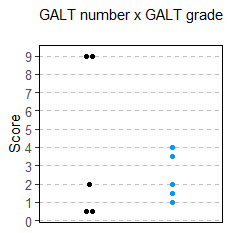

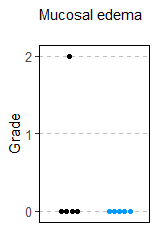

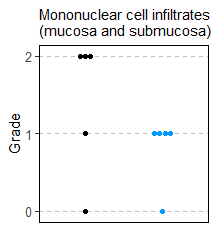

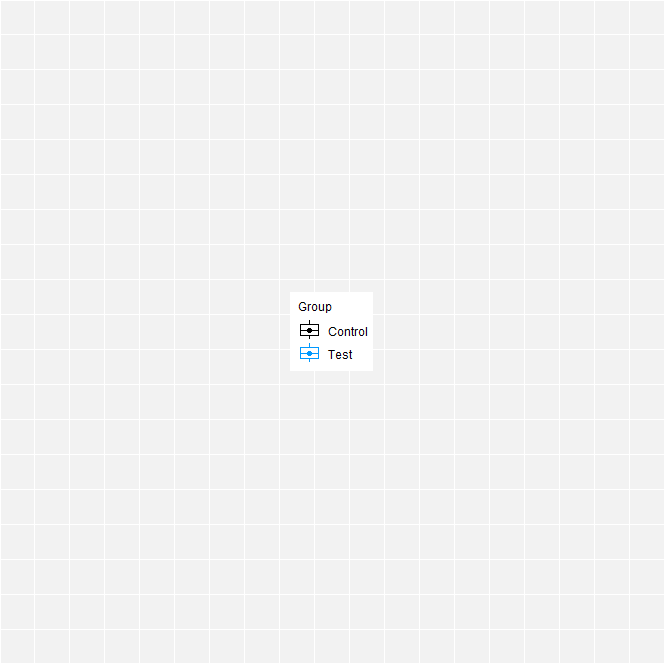
**
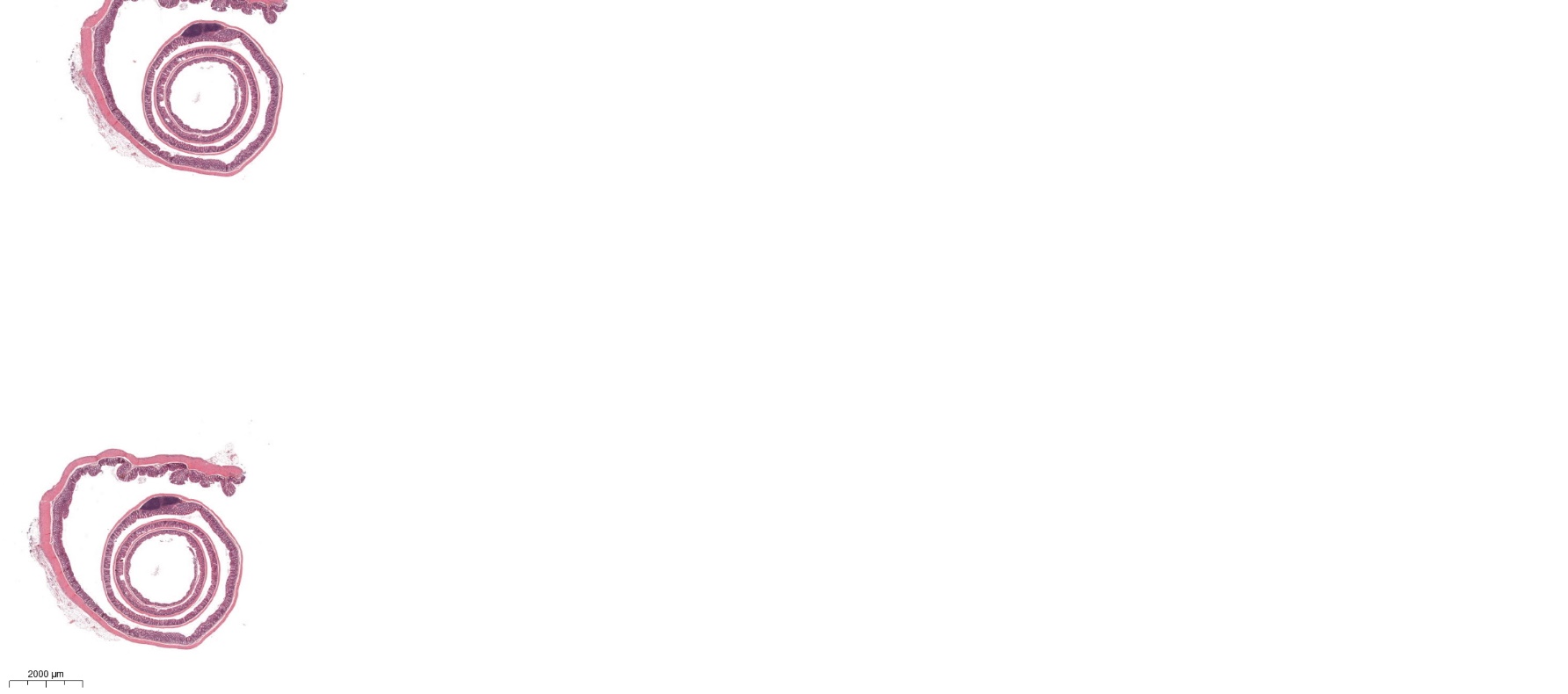

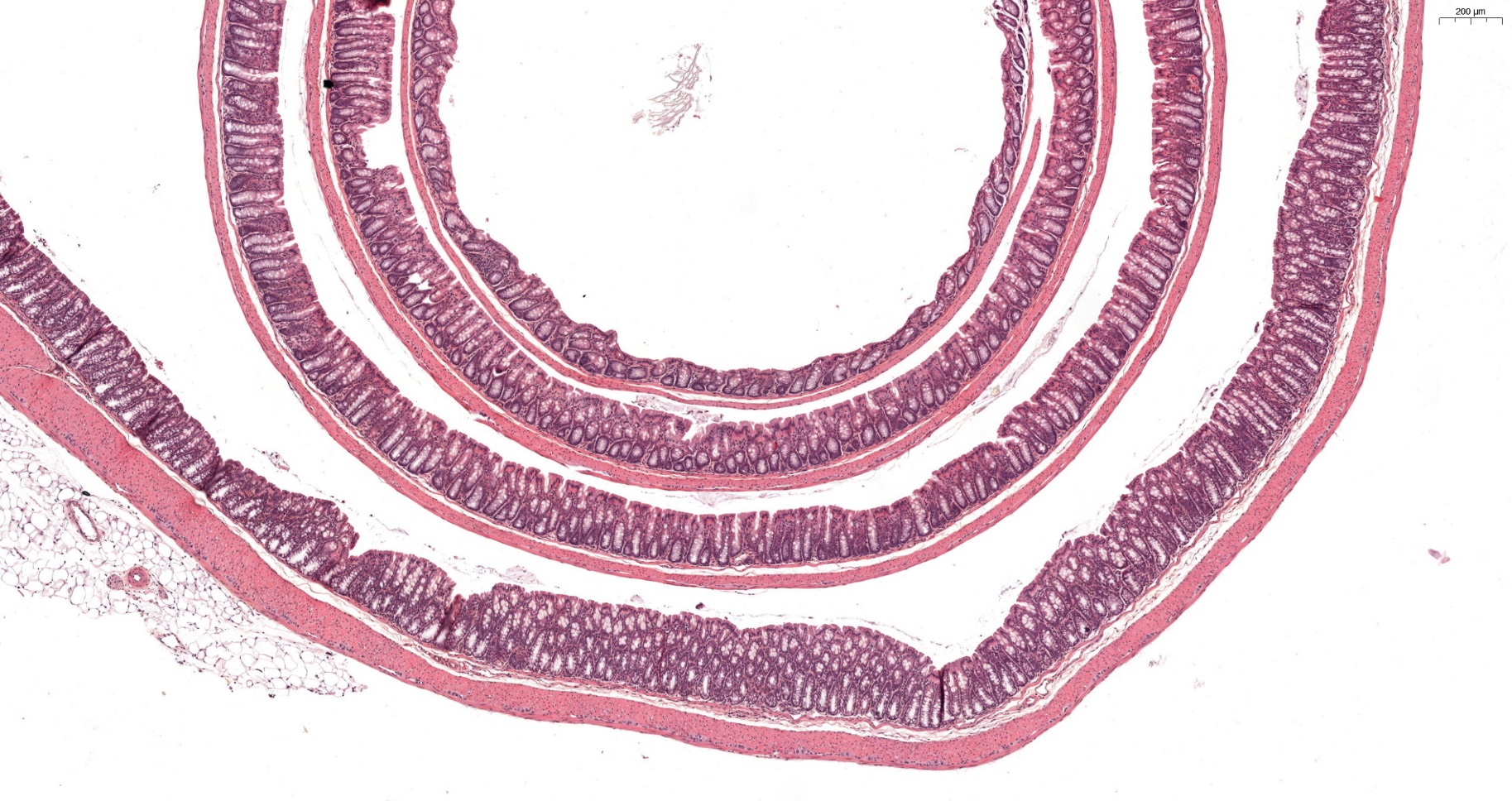

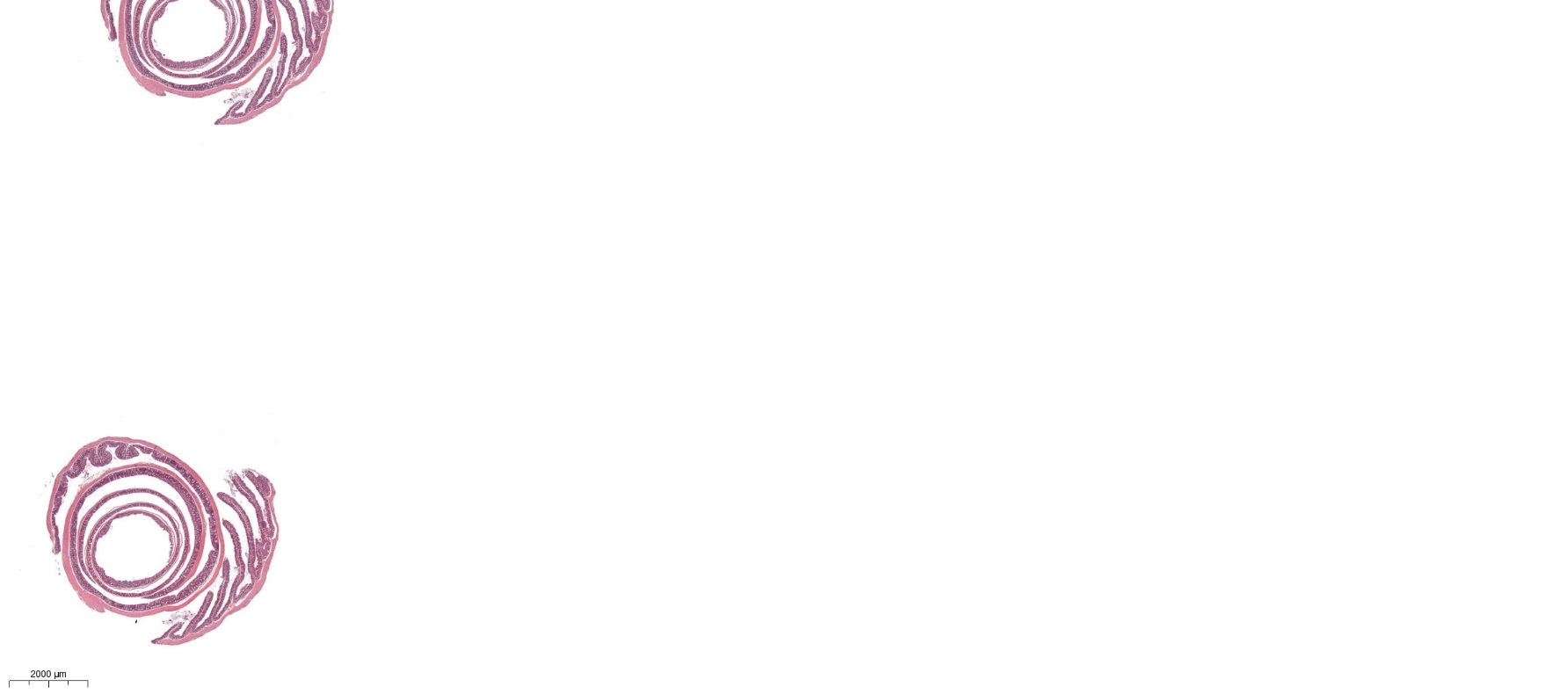

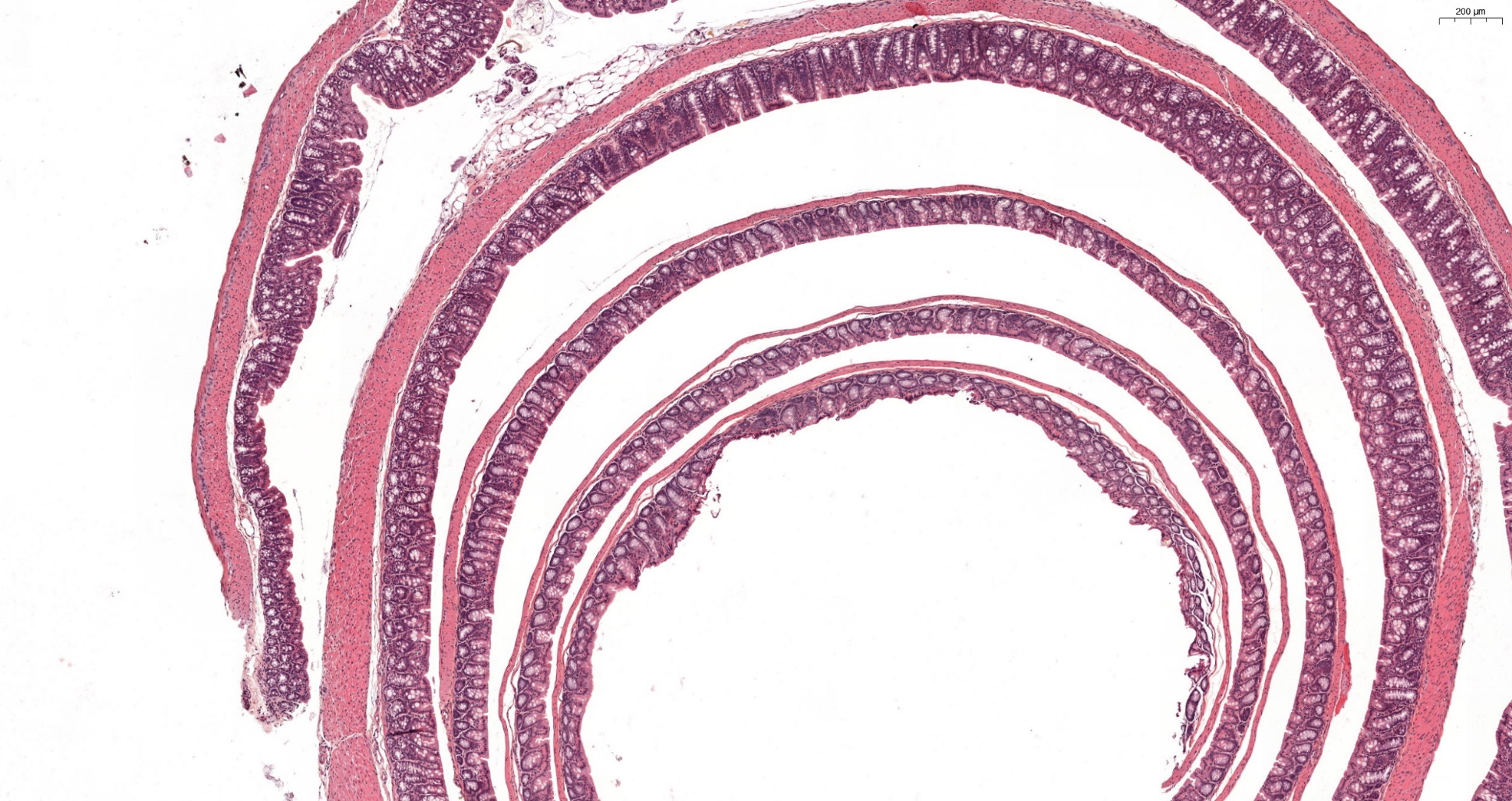
**

**Supplementary Fig. S11. Histo-pathological scores and pictures of colon swiss rolls at the end of the experiment.** Only criteria with scores > 0 are shown. No statistically significant differences were observed between groups (Fisher-Pitman permutation test). The two snapshots on the left are from a mouse in the control group and those on the right from the test group.


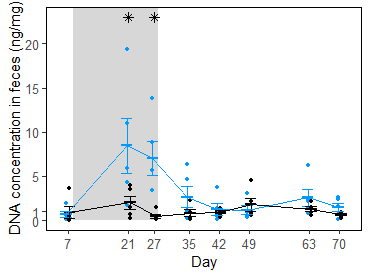

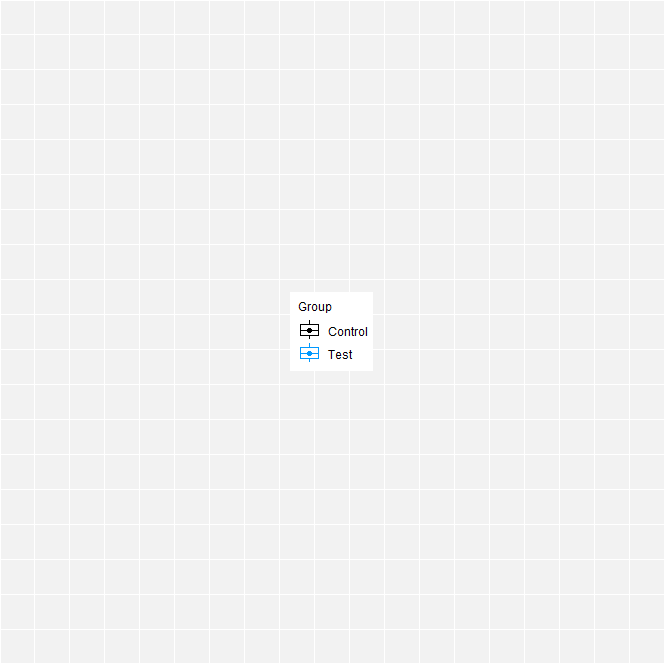


**Supplementary Fig. S12. Longitudinal quantification of host DNA in feces.** Measured by qPCR targeting LINE-1 repeats. **⚹**: p<0.05, Fisher-Pitman permutation test. The grey area represents the RFD.


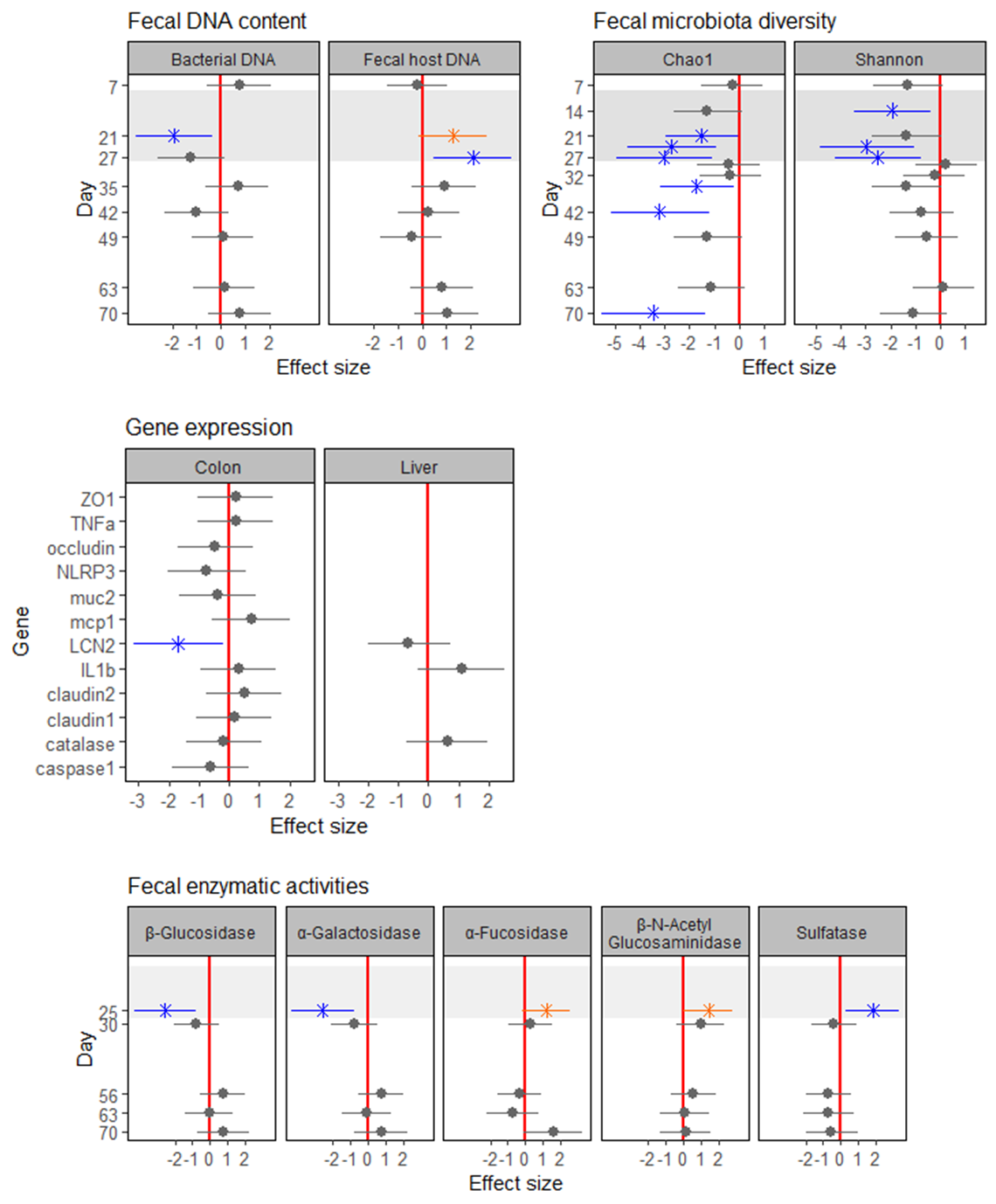


**Supplementary Fig. S13. Summary of all measurements and effect sizes (test group *vs* control group).** The effect size was calculated using Cohen's d and is represented with a 95% confidence interval. The grey area on the “Days” axis represents the RFD (for the test group). The stars indicate factors that are statistically different between groups with a Fisher-Pitman permutation test. In blue are factors for which the effect size is > 0.8 and the confidence interval of the effect size does not contain 0. In orange are factors for which the confidence interval of the effect size contains 0 but are statistically different with a permutation test.

**Supplementary Table S1.** Diet compositions.

**Supplementary Table S2.** Sequencing facilities details

**Supplementary Table S3.** List of ASVs with significantly different abundances between groups at the end of RFD (day 27, p-value from Wilcoxon test <0.05, no correction), along with their median abundance in each group, median differences between and within groups, effect size and taxonomic affiliation.

**Supplementary Table S 4.** List of ASVs with significantly different abundances between groups at the end of the experiment (day 70, p-value from Wilcoxon test <0.05, no correction), along with their median abundance in each group, median differences between and within groups, effect size and taxonomic affiliation.

**References used in the supplementary material**

1. Fernandes, A. D., Macklaim, J. M., Linn, T. G., Reid, G. & Gloor, G. B. ANOVA-Like Differential Expression (ALDEx) Analysis for Mixed Population RNA-Seq. *PLOS ONE* **8**, e67019 (2013).

2. National Center for Biotechnology Information (NCBI). Accession No. NR_181880.1. Sangeribacter muris strain A43 16S ribosomal RNA, partial sequence. (2022).

3. National Center for Biotechnology Information (NCBI). Accession No. NR_170509.1. Duncaniella freteri strain TLL-A3 16S ribosomal RNA, partial sequence. (2020).

4. National Center for Biotechnology Information (NCBI). Accession No. NR_181879.1. Duncaniella muricolitica strain A60 16S ribosomal RNA, partial sequence. (2022).

5. National Center for Biotechnology Information (NCBI). Accession No. NR_144616.1. Muribaculum intestinale strain YL27 16S ribosomal RNA, partial sequence. (2019).

6. National Center for Biotechnology Information (NCBI). Accession No. NR_179975.1. Duncaniella muris strain 129-Nlrp6 16S ribosomal RNA, partial sequence. (2022).

7. National Center for Biotechnology Information (NCBI). Accession No. NR_170508.1. Duncaniella dubosii strain H5 16S ribosomal RNA, partial sequence. (2020).

8. National Center for Biotechnology Information (NCBI). Accession No. NR_179976.1. Paramuribaculum intestinale strain B1117 16S ribosomal RNA, partial sequence. (2022).

9. Paradis, E. & Schliep, K. ape 5.0: an environment for modern phylogenetics and evolutionary analyses in R. *Bioinformatics* **35**, 526–528 (2019).
